# Human beige adipocytes for drug discovery and cell therapy in metabolic diseases

**DOI:** 10.1038/s41467-020-16340-3

**Published:** 2020-06-02

**Authors:** Amar M. Singh, Liang Zhang, John Avery, Amelia Yin, Yuhong Du, Hui Wang, Zibo Li, Haian Fu, Hang Yin, Stephen Dalton

**Affiliations:** 1 Department of Biochemistry and Molecular Biology, Center for Molecular Medicine, University of Georgia, Athens, GA 30602 USA; 2 Department of Pharmacology and Chemical Biology, Emory Chemical Biology Discovery Center, Emory University School of Medicine, Atlanta, GA 30322 USA; 3 Department of Radiology, Biomedical Research Imaging Center, University of North Carolina Chapel Hill, Chapel Hill, NC 27599 USA; 4 Department of Hematology and Medical Oncology and Winship Cancer Institute, Emory University School of Medicine, Atlanta, GA 30322 USA

**Keywords:** Differentiation, Mesenchymal stem cells

## Abstract

Human beige adipocytes (BAs) have potential utility for the development of therapeutics to treat diabetes and obesity-associated diseases. Although several reports have described the generation of beige adipocytes in vitro, their potential utility in cell therapy and drug discovery has not been reported. Here, we describe the generation of BAs from human adipose-derived stem/stromal cells (ADSCs) in serum-free medium with efficiencies >90%. Molecular profiling of beige adipocytes shows them to be similar to primary BAs isolated from human tissue. In vitro, beige adipocytes exhibit uncoupled mitochondrial respiration and cAMP-induced lipolytic activity. Following transplantation, BAs increase whole-body energy expenditure and oxygen consumption, while reducing body-weight in recipient mice. Finally, we show the therapeutic utility of BAs in a platform for high-throughput drug screening (HTS). These findings demonstrate the potential utility of BAs as a cell therapeutic and as a tool for the identification of drugs to treat metabolic diseases.

## Abstract (web summary)

Methods to generate beige adipocytes from a human cell source are inefficient. Here, the authors present a protocol that efficiently generates beige adipocytes from human adipose-derived stem cells (ADSCs), which have potential utility in therapeutic development relating to metabolic diseases such as type 2 diabetes.

## Introduction

Current medications for type 2 diabetes (T2D) are neither preventative, nor curative, and only serve to manage the disease^1^. Most medications such as metformin, dipeptidyl-peptidase inhibitors, and meglitinides, function mainly to inhibit glucose production or stimulate insulin secretion^2^, and are used as long-term treatments. Owing to drug efficacies and disease severity, diabetics often require combination drug therapies, including injectable insulin, resulting in complicated drug regimens and long-term management care programs. Therefore, there is a clear need to develop new pharmaceuticals or cell-based therapies that could reduce or prevent this disease burden.

Thermogenic (brown, beige/brite) adipocytes have therapeutic potential for the treatment of obesity-associated diseases, such as T2D^3–5^ based on their ability to modulate circulating glucose levels. This is supported by animal model experiments showing that transplantation of mouse and human brown or beige adipocytes reduce obesity and hyperglycemia in recipients^6–11^. These proof-of-concept experiments are further supported by clinical observations, demonstrating an inverse correlation between body mass index and the amount of brown fat depots in the body^12–16^. Together, these observations indicate that thermogenic adipocytes are an important determinant of metabolic disease, including T2D.

Thermogenic adipocytes are distinguished from white adipocytes (WA) by their high mitochondrial mass and uncoupling activity^3^. Unlike WA, that serve as a storage depot for triglycerides, thermogenic adipocytes oxidize fatty acids and generate heat through uncoupled mitochondrial respiration, thereby impacting non-shivering thermogenesis. Moreover, thermogenic adipocytes modulate circulating glucose and triglyceride levels and use these to fuel heat generation. Thermogenic adipocytes are stimulated by norepinephrine-dependent activation of β-adrenergic signaling. In the mouse, β3 receptor-dependent signaling is required for thermogenic activation^17^. This signaling pathway functions through cyclic-AMP (cAMP)/protein Kinase-A (PKA), which induces lipolysis and uncoupling protein-1 (UCP1) activity, resulting in elevated uncoupled respiration and thermogenesis.

It has been proposed that beige adipocytes could have utility as a cell therapeutic for treating T2D by serving to normalize circulating blood glucose levels. One of the main obstacles preventing the use of thermogenic adipocytes in a cell therapy setting, however, is the availability of a sufficiently pure cell source with functional characteristics that enable translational development^5^. Although several reports have described the generation of brown and beige adipocytes from human pluripotent stem cells^10,18–21^, none of these reports demonstrate utility in clinical development because of inefficient differentiation, genetic modifications, and/or lack of in vivo functional validation. Two previous reports^22,23^ describe the generation of beige cells from human ADSCs but these methods are inefficient and generate cell populations of uncharacterized composition. Moreover, in vivo activity of these cells has not been previously been reported. A functional, defined source of beige adipocytes would also have significant potential for the development of new pharmacological and natural product-based therapeutics in the T2D and obesity areas^4,5,24^. The “cell source” problem described above however, has severely limited this approach^25^.

In this report, we describe a method for the generation of beige adipocytes from adult human ADSCs with high efficiency in a fully defined, serum-free medium. This approach was suggested by preliminary studies, indicating that ADSCs may have the potential for beige adipocyte differentiation^22,23^, although the efficiency and reproducibility of these findings have been controversial^26^. These early studies also failed to demonstrate functional activity^22,23^ in vivo, making their clinical potential unclear. Beige adipocytes described in this report exhibit a molecular profile similar to brown adipocytes isolated from human tissue and have metabolic activity characteristic of thermogenic adipocytes, including uncoupled respiration and cAMP-induced lipolysis^17^. Following transplantation into recipient mice, ADSC-derived beige adipocytes show robust glucose uptake and elevated thermogenic activity while impacting body weight. A high-throughput screening (HTS) for small molecules that activate lipolysis in beige adipocytes demonstrates their utility in drug-screening applications. It also points towards a pathway for the development of a new category of therapeutics to treat metabolic diseases, including T2D and obesity, with potential applications in disease treatment and prevention.

## Results

### Differentiation of ADSCs and characterization of beige adipocytes

**Fig. 1:**
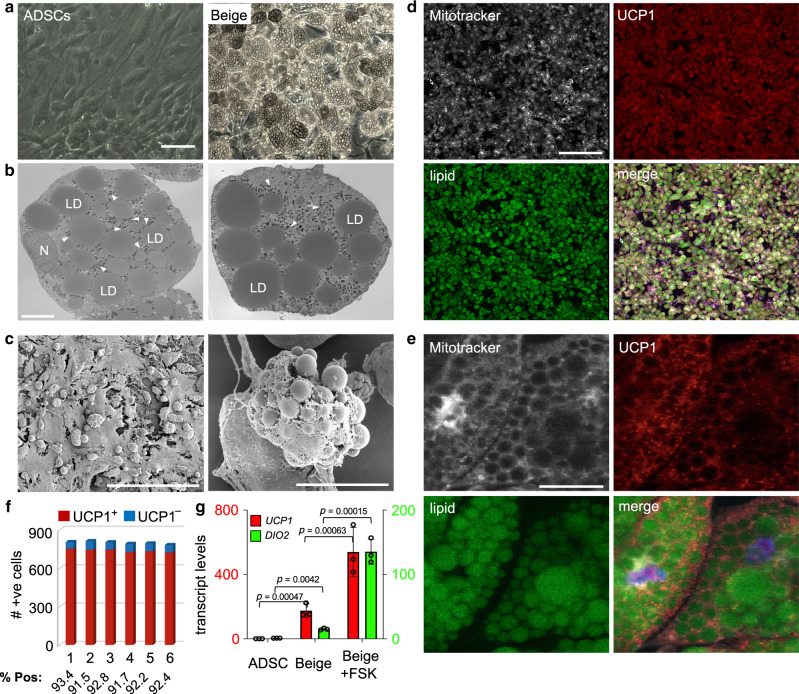
Efficient generation of beige adipocytes from ADSCs. **a** Phase-contrast images of ADSCs and beige adipocytes, bar 100 μm. **b** Transmission electron microscopy of ADSC-derived beige adipocytes, two independent fields of view are shown. LD, lipid droplets; N, nucleus, arrowheads, mitochondria. Bar, 6 μm. **c** Scanning electron microscopy of beige adipocytes grown in culture. Left, bar 300 μm; Right, bar, 30 μm. **d**, **e** Immunostaining of beige adipocytes for UCP1, along with LipidTOX green (lipid) and MitroTracker Deep Red (mitochondria), bar 300 μm for **d** and 50 μm for **e**. **f** Quantitation of immunostaining data from six independent fields of view, with >780 cells counted/field. **g** *UCP1* and *DIO2* transcript levels were determined by qRT-PCR in ADSCs and ADSC-derived beige adipocytes ± forskolin, (FSK, 20 μM, 6 hours). Data are presented as mean ± S.D. and representative of three biologically independent replicates. *p* values were calculated by unpaired two-tailed Student’s *t* test.

ADSCs used in this study were characterized and validated by analysis of cell surface markers (Supplementary Fig. 1a) and RNA-seq analysis (Supplementary Fig. 1b). This analysis confirmed that cells used in this study are comparable to equivalent cells reported in the literature. To obtain a highly enriched beige adipocyte population from human ADSCs, a chemically defined media was developed by testing factors individually or combinatorially for pro-thermogenic activity in basal medium^27–30^ (Supplementary Fig. 2, Supplementary Fig. 3 and Supplementary Table 1). The final beiging medium (B-8) used for conversion of ADSCs to beige adipocytes consisted of eight growth factors and inhibitors that directed high-efficiency differentiation over 21 days. Resulting cells exhibited clusters of lipid droplets surrounded by an abundance of mitochondria (Fig. 1a–c) and elevated expression of mitochondria-localized UCP1 (Fig. 1d, e). More than 90% of cells generated by culture of ADSCs in B-8 media express UCP1 and are LipidTox Green^+^ after 21 days (Fig. 1f, Supplementary Fig. 4). The B-8 method was compared with the previously described methods by Bartesaghi et al.^24^ and Wang et al.^25^ (Supplementary Fig. 5). Less than 30% of cells generated by these latter approaches expressed UCP1, indicating that the majority of cells in these cultures are not beige adipocytes. Cells showed clear temporal changes in the expression of general adipocyte markers under B-8 differentiation conditions followed by increased levels of *UCP1* mRNA, consistent with them transitioning from a general pre-adipocyte state to a thermogenic, beige adipocyte state (Supplemental Fig. 6). The efficacy of beige cell differentiation with B-8 medium was confirmed using six, independent human ADSC primary cell lines. Efficient differentiation of ADSCs to a beige state occurred independently of passage number, gender of the donor or, body mass index and T2D status of donors (Supplementary Figs. 7 and 8).

To establish if ADSC-derived beige adipocytes are responsive to signaling pathways required for the activation of thermogenic adipocytes, cells were treated with forskolin (FSK) to activate adenyl cyclase and intracellular cAMP levels^31,32^. In the resting state, beige adipocytes express 170- and 15-fold higher levels of *UCP1* and *DIO2* transcripts, respectively, compared with ADSCs (Fig. 1g). Stimulation with FSK, further increased levels of *UCP1* and *DIO2* transcripts by 520- and 130-fold compared with ADSCs, respectively (Fig. 1g). These observations are consistent with the anticipated response of bona fide thermogenic adipocytes to activated cAMP-dependent signaling^33^.

**Fig. 2:**
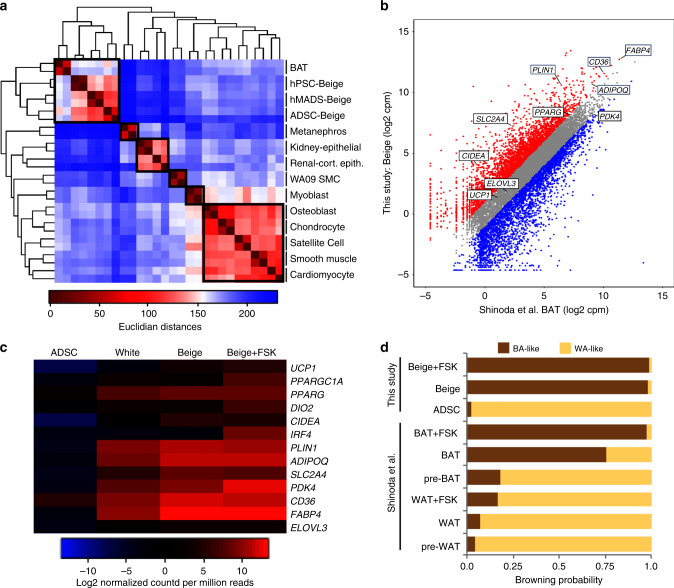
Global transcript analysis of ADSC-derived beige adipocytes. **a** Hierarchical clustering dendrogram comparing ADSC-derived beige adipocytes to other primary human cell types. Boxes indicate cell types with similar Euclidian distances. **b** Scatter plot comparing global transcriptomes of ADSC-derived beige adipocytes and human brown adipocytes^33^. Transcripts typically expressed in thermogenic adipocytes at elevated levels are indicated. Gray data points represent less than twofold difference between data sets, red data points represent less than twofold increase in this study and blue data points represent less than twofold increase in brown adipocytes. **c** Heatmap of RNA-seq data focusing on transcripts typically enriched in thermogenic adipocytes. ADSCs, white adipocytes, and ADSC-derived beige adipocytes ± forskolin, FSK (20 μm, 6 hours) are shown. **d** ProFAT^37^ computational analysis from reference data sets that integrates 97 human brown and white adipocyte samples, compared with the RNA-seq from ADSC-derived beige adipocytes. The browning probability score was determined from the correlation between the reference data sets and the ADSC-derived beige adipocyte data set. Data for beige, white (WA), and brown (BA) adipocytes were analyzed through this pipeline.

Hierarchical clustering analysis of RNA-seq data show that ADSC-derived beige adipocytes cluster closely with other human thermogenic adipocytes, including human brown^33^ and beige^26^^,^^34,35^ adipocytes. These different sources of thermogenic adipocytes segregate away from other human cell types included in this analysis^36^ (Fig. 2a). Moreover, comparing global gene expression signatures in beige and brown adipocytes showed a high correlation under unstimulated and FSK-treated conditions (Fig. 2b and Supplementary Fig. 9a–c). Beige adipocytes exhibit elevated levels of thermogenic markers, compared with that in WA and ADSCs (Fig. 2c). In addition, levels of these thermogenic adipocyte marker were upregulated in beige cells following induction with FSK (Fig. 2c). Finally, we calculated the “browning probability score” using ProFAT, a recently developed computational assessment tool^37^, that combines 97 human adipose microarray and RNA-seq data sets from various sample types to identify a common expression signature for white and brown adipocytes. The brown adipocyte signature identified by ProFAT analysis can then be used to derive a brown adipocyte correlation value that is an indicator of brown adipocyte identity. When RNA-seq data from ADSC-derived beige cells was applied to ProFAT, a browning probability coefficient of 0.98 was obtained (Fig. 2d and Supplementary Fig. 10), indicative that these cells are thermogenic adipocytes. This correlation value exceeds that assigned to human brown adipocytes derived from immortalized pre-adipocytes^33^ (Fig. 2d). The phenotypic and molecular characteristics of these cells are consistent with authentic beige adipocytes. These data collectively establish this method as a robust platform to generate ADSC-derived beige adipocytes.

### Metabolic analysis of human beige adipocytes in vitro

**Fig. 3:**
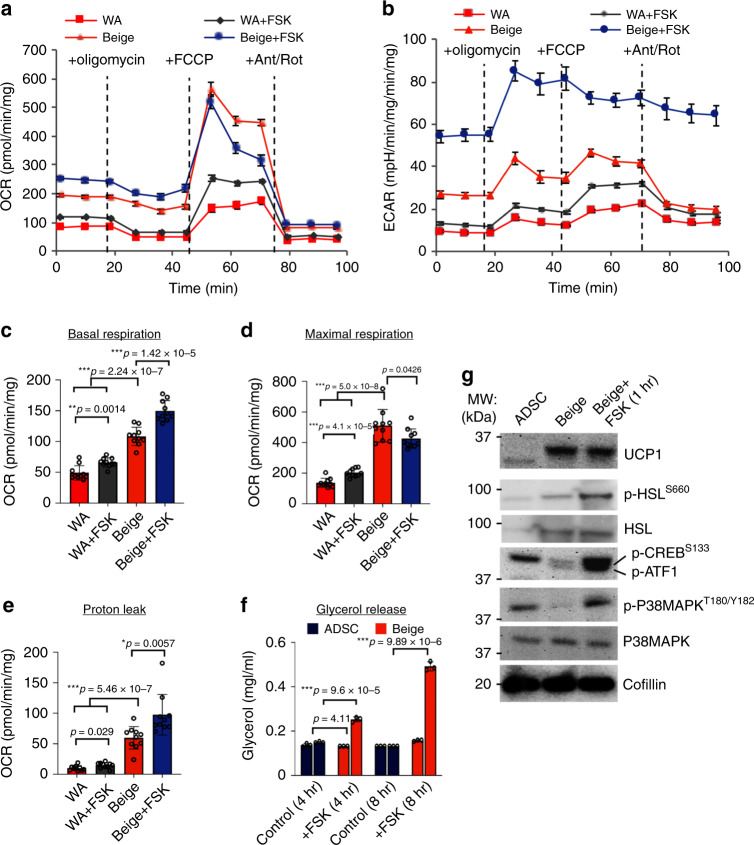
Metabolic activity of ADSC-derived beige adipocytes in vitro. **a** Oxygen consumption rates (OCR) following the Seahorse Mito Stress Test assay in beige adipocytes compared with white adipocytes (WA). Cells (ASC52telo) were stimulated with forskolin (FSK, 20 μM) for 24 hours prior to treatment, and oligomycin (3 μM), FCCP (3 μM) and antimycin A/rotenone (5 μM each) were added at indicated time points. Assays were normalized to total protein content. **b** Extracellular acidification rates (ECAR) of samples in **a**. **c**–**e** Basal respiration rate, maximal respiration rate and proton leak calculated from Seahorse assay performed in **a**. **f** Lipolysis assay for ADSCs and ADSC-derived beige adipocytes, either untreated or treated with FSK for the indicated times. **g** Immunoblotting of whole-cell lysates (25 μg) from ADSCs and ADSC-derived beige adipocytes ± FSK (1 hr, 20 μM). All data are presented as mean ± S.D. and representative of 10 biologically independent replicates. *p* values were calculated by unpaired two-tailed Student’s *t* test.

The ability of ADSC-derived beige adipocytes to undergo elevated rates of uncoupled mitochondrial respiration and fatty-acid oxidation under basal and stimulated (+FSK) conditions was evaluated. Oxygen consumption rates (OCR) and extracellular acidification rates (ECAR) were used as readouts to determine respiratory and glycolytic rates, respectively (Fig. 3a, b). These analyses showed that beige adipocytes have significantly higher basal (Fig. 3a, c) and maximal (Fig. 3a, d) rates of mitochondrial respiration as well as significantly higher rates of glycolysis (Fig. 3b) compared with human ADSC-derived WA (Fig. 3a, b). As expected, the mitochondrial respiration and glycolytic responsiveness of beige adipocytes to FSK was significantly greater than that for WA (Fig. 3a, b). Basal and maximal respiration rates were also significantly elevated in beige cells compared with white adipocytes (Fig. 3c, d). Most importantly, the basal level of uncoupled respiration (proton leak) was fivefold higher in beige adipocytes compared with WA. Following stimulation with FSK, uncoupled respiration increased by 64.7% relative to basal conditions (Fig. 3e). These data demonstrate that beige adipocytes are metabolically distinct from WA in vitro and have a metabolic capacity for elevated glucose oxidation and uncoupled respiration, similar to that described for thermogenic adipocytes in vivo^33^.

In addition to the activation of thermogenesis through impacting adenylate cyclase activity, FSK activates lipolysis in brown and beige adipocytes^31,32^. To establish if increased adenyl cyclase activity activates lipolysis in ADSC-derived beige adipocytes a glycerol release assay was performed. Following addition of FSK a ~2 and 5-fold increase in free glycerol was detected in the culture medium after 4 and 8 hours, respectively. This confirms that lipolytic activation mechanisms acting through cAMP are intact in ADSC-derived beige cells (Fig. 3f). Previous studies have demonstrated that FSK induces cAMP and PKA activity to promote lipolysis^38^ through a mechanism requiring the phosphorylation of cAMP-response element binding protein (CREB) and P38MAPK activation^39,40^. Increased cAMP levels and PKA activation also activate hormone-sensitive lipase (HSL) by a phosphorylation-dependent mechanism^41^. Activation of HSL is an important determinant of lipolysis in thermogenic adipocytes. To confirm that beige adipocyte activation by FSK occurs by a canonical thermogenic mechanism, HSL, CREB, and P38MAPK are shown to be phosphorylated within 1 hour of FSK-treatment (Fig. 3g). This confirms that well-established pathways required for beige adipocyte activation are functional in ADSC-derived beige adipocytes. Overall, these data indicate that ADSC-derived beige adipocytes are activated by the cAMP-adenyl cyclase signaling axis, resulting in increased uncoupled respiration, glucose utilization and lipolysis.

### Beige adipocytes modulate metabolic activity in vivo

**Fig. 4:**
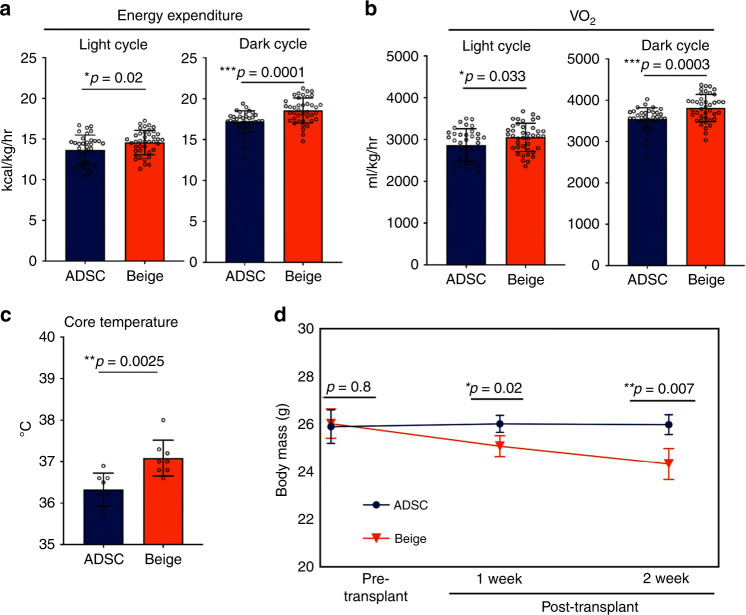
Beige adipocyte transplantation increases energy expenditure, oxygen consumption, and reduces body weight and hyperglycemia. **a** NOD/SCID mice were transplanted with two million ADSCs or ADSC-derived beige adipocytes and assayed by indirect calorimetry. *n* = 7 control, *n* = 8 beige cells. Average measurements of energy expenditure for light and dark cycles are indicated. **b** Oxygen consumption levels (VO_2_) from indirect calorimetry assays. **c** Core body temperature measurements in ADSC and beige adipocyte-transplanted mice, *n* = 4 per group. **d** Body mass determination in ADSC and beige adipocyte-transplanted mice, *n* = 4 per group. All data are presented as mean ± S.D. *p* values were calculated using the Mann–Whitney rank sum tests for indirect calorimetry and unpaired two-tailed Student’s *t* test for body mass and body temperature.

Results presented here demonstrate that beige adipocytes can be generated at high efficiency from ADSCs and that they are phenotypically, genotypically, and metabolically comparable to thermogenic adipocytes derived from BAT^42^. To establish that beige adipocytes impact metabolic regulation in vivo, their activity following transplantation was evaluated. Following transplantation of beige adipocytes and ADSCs into the hindlimb muscle of NOD/SCID, indirect calorimetry was performed to evaluate whole-body energy expenditure (EE). It is predicted that following transplantation, authentic thermogenic adipocytes would increase total EE as a consequence of their elevated rates of glucose consumption, fatty-acid oxidation and uncoupled respiration. Using metabolic cage assays, increased whole-body energy and oxygen consumption was confirmed in recipients receiving beige cells but not ADSCs (Fig. 4a, b). Importantly, no changes in respiratory exchange ratios or locomotor activities (beam breaking) were observed between the two groups, indicating that increased EE and O_2_ consumption in mice transplanted with beige cells was not owing to variations in locomotive activity (Supplementary Fig. 11a, b). Mice receiving beige adipocytes showed a ~2% increase (0.8 °C, *p* < 0.005) in core body temperature relative to ADSC recipients (Fig. 4c), indicating that beige adipocyte-transplanted mice expended significantly more energy than their ADSC-transplanted counterparts (Fig. 4a). Consistent with this, surface body temperatures of mice receiving beige adipocytes were significantly increased over those receiving ADSCs, as determined by thermal imaging (Supplementary Fig. 12). A significant reduction in body weight was also observed in mice receiving beige adipocytes compared with ADSC recipients over a 2-week period (Fig. 4d). These data provide evidence that transplanted ADSC-derived beige adipocytes increase EE resulting in elevated heat generation and general metabolic activity.

**Fig. 5:**
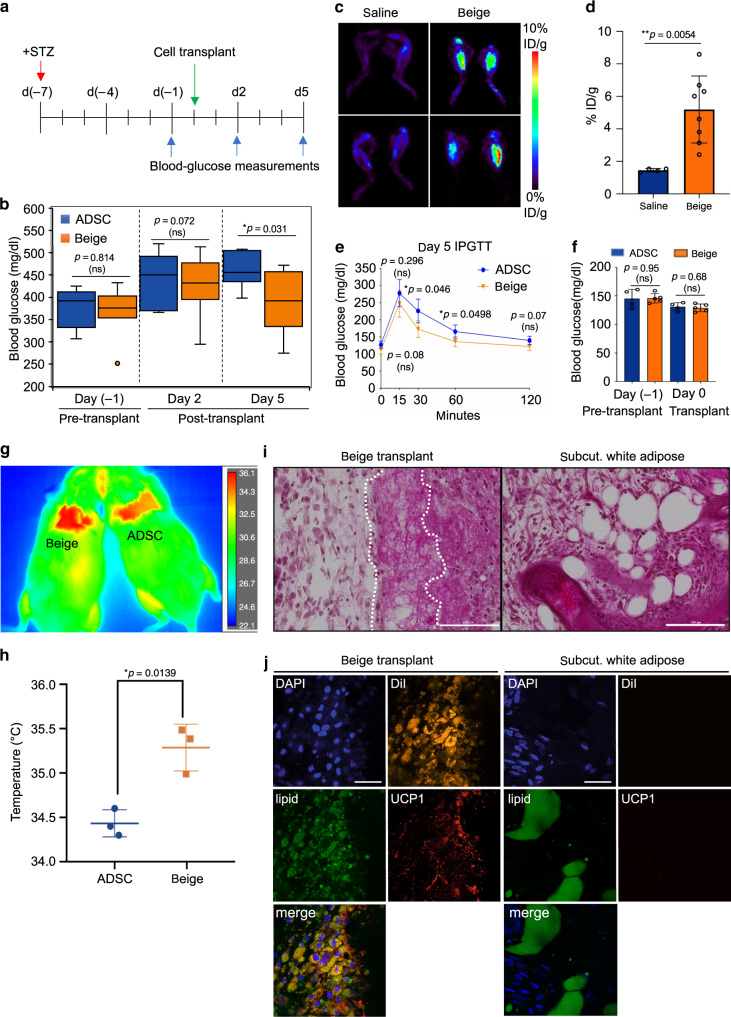
Engrafted human beige adipocytes increase glucose clearance and heat generation. **a** Experimental design for streptozotocin (STZ)-induced hyperglycemia. Times of STZ administration (red arrow), cell transplantation (green arrow), and blood glucose sampling (blue arrow) are indicated. **b** Box-plot showing quartile distribution and a median line of blood glucose measurements in STZ-treated mice from **a**. *n* = 8 per group. **c** Representative images of PET scans following treatment of mice with ^18^F-FDG. Data is graphed as the % injected dose/gram body mass (% ID/g) at 2-weeks post transplantation of beige adipocytes compared with saline, *n* = 4 per group. **d** Quantitation of PET analysis, *n* = 4 per group. **e** Intraperitoneal glucose tolerance test (IPGTT) following transplantation (5 days) into the intrascapular, hindlimb regions, *n* = 5 per group. **f** Pre-transplant blood glucose levels for data shown in **e**, *n* = 5 per group. **g** Representative infrared image of mice transplanted into the intrascapular region with human beige adipocytes or human ADSCs. **h** Plots of thermal imaging data as in **g** ±standard deviation, *n* = 3, per group. **i** Histological analysis of engrafted human beige adipocytes (left) compared with subcutaneous white adipocyte depot (right) by H&E staining at day 5, 100 μm. **j** Immunohistochemistry of 10 μm frozen sections. Left: sections from tissue transplanted with DiI-labeled beige adipocytes, 5 d post transplantation. Right: adjacent region to transplant of subcutaneous white adipose. Sections were probed with antibodies for UCP1, LipidTox Green, DAPI. Micron bar: 30 μM. All data are presented as mean ± S.D. and *p* values were calculated by unpaired two-tailed Student’s *t* test.

To examine if hADSC-derived beige adipocytes can metabolically function in vivo, their ability to impact levels of circulating blood glucose was determined using a chemically induced (streptozotocin, STZ), hyperglycemic mouse model. Male NOD/SCID mice were treated with STZ and then transplanted with either ADSCs or beige cells into the hindlimb muscle (Fig. 5a). Over the time-course of this experiment, beige cells significantly reduced the levels of circulating glucose in hyperglycemic mice compared with ADSC controls (*p* < 0.05) (Fig. 5b). To confirm that transplanted beige cells engraft and actively clear circulating glucose, ^18^F-fluorodeoxyglucose (FDG) small animal positron emission tomography (PET) was performed (Fig. 5c, d). Following administration of a bolus of ^18^F-FDG, a ~3.5-fold increase in ^18^F-FDG uptake was observed in mice transplanted with beige cells compared with controls (*p* < 0.05). This was supported by intraperitoneal glucose tolerance tests where mice transplanted with beige adipocytes cleared circulating glucose significantly more quickly (*p* < 0.05) than ADSC controls (Fig. 5e, f). Differences in food intake (Supplementary Fig. 13) or potential “browning” of white adipose depots (Supplementary Fig. 14) do not account for changes in metabolic activity observed between beige and ADSC-recipient mice. Under thermoneutral conditions, glucose clearance in beige cell recipients was not statistically different to ADSC controls, although a positive trend was noted (Supplementary Fig. 15). This indicates that transplanted beige cells are likely to be under similar physiological regulatory cues that regulate thermogenic adipocytes in vivo^43^.

**Fig. 6:**
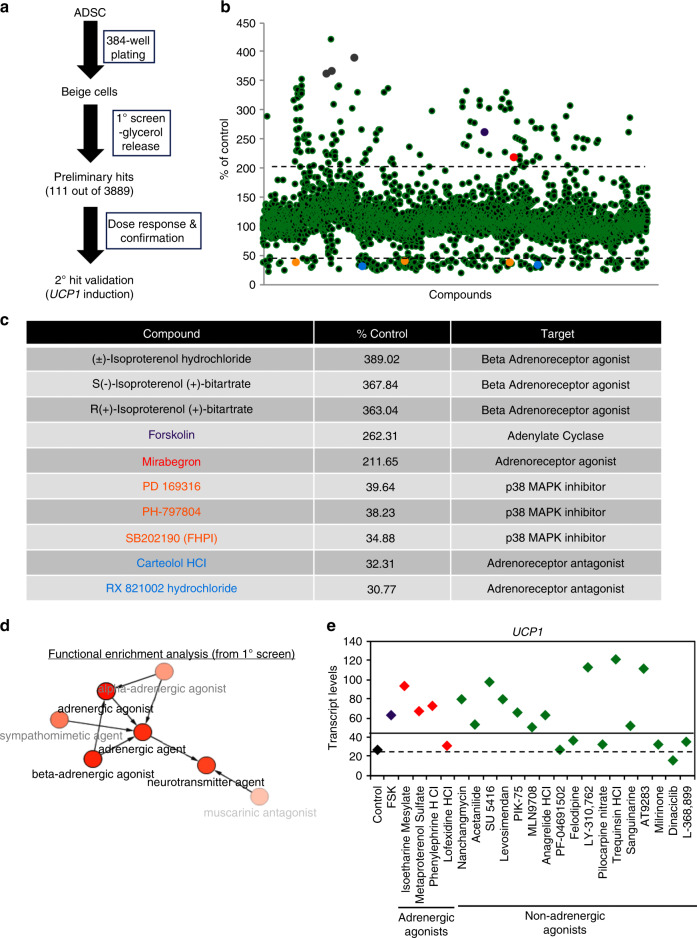
High-throughput screening for small molecule activators of beige adipocytes. **a** Scheme used for high-throughput screening (HTS). **b** Scatter plot of glycerol release for each compound assayed (*n* = 3889) in the HTS screen. Data are shown as the percentage of glycerol released compared with untreated beige adipocytes (% of control). **c** Representative compounds (color indicates location in scatter plot **b**) are shown with relative levels of glycerol release (% control) and reported targets for these compounds. **d** Computational analysis of preliminary hits identified in the HTS using BiNChE^45^. **e** Secondary hit validation of primary compounds was performed by evaluating levels of *UCP1* transcript by qRT-PCR. Unstimulated beige cells (black), forskolin (purple), selected adrenergic agonists (red), and potential new non-adrenergic beige fat activators identified in the primary screen are shown. Transcript levels were normalized to *UCP1* levels in ADSCs, *n* = 3 for each technical replicate. Dashed line is the ADSC background signal level and the solid line is the twofold activation threshold.

Increased glucose clearance in beige adipocyte recipients was accompanied by increased thermogenic activity (Fig. 5g, h). Histological analysis of tissue isolated from these mice confirmed the presence of adipocytes with multilocular lipid droplets, consistent with the presence of beige adipocytes (Fig. 5i, “beige transplants”). This morphology is clearly different to subcutaneous WA depots from the same mouse (Fig. 5i, “subcut. white adipose”). Immunohistochemistry analysis of the transplanted (DiI^+^) tissue confirmed the engraftment of human UCP1^+^ cells with multilocular lipid droplets (Fig. 5j, left panel). As anticipated, human UCP1^+^ cells were absent in subcutaneous white adipose depots (Fig. 5j, right panel). These data demonstrate that human ADSC-derived beige cells engraft into recipient mice and exhibit metabolic activity consistent with them being functional beige adipocytes.

### A high-throughput beige adipocyte drug-screening platform

The identification of new drugs and regulatory pathways that promote the activation of endogenous thermogenic adipocytes may result in the development of new therapeutics for the treatment of metabolic diseases, such as diabetes and/or obesity. To test the utility of beige adipocytes for drug discovery, the LOPAC and Emory Enriched Bioactive Library^44^ (Fig. 6a) were screened using a lipolysis assay (glycerol release) in a 384-well plate format.

Out of 3889 compounds evaluated in the primary screen, 111 compounds significantly stimulated glycerol release and were subject to further evaluation (Fig. 6b). Importantly, well-known activators of thermogenic adipocytes including FSK and β-adrenergic agonists such as isoproterenol were identified as primary hits in this screen (Fig. 6b, c), confirming the utility of the assay. Inhibitors of p38 MAPK and adrenoreceptors that suppress lipolysis were also identified^29–31^ (Fig. 6b, c). The chemical enrichment analysis tool, BiNChE^45^ was then used to identify mechanistic links between the 111 preliminary hits identified in this screen. As anticipated, adrenergic signaling was identified as a key regulatory pathway for beige adipocyte activation in the screen (Fig. 6d). Thirty-six of the primary screen hits are known adrenergic agonists, indicating that the screening platform is reliably detecting authentic activators of beige adipocytes. Next, we performed lipolysis assays for all 111 compounds identified in the primary screen at three different concentrations, of which 24 were selected for further analysis based on their novelty and/or reproducibility (Supplementary Data 1). All 24 compounds significantly increased lipolysis in the three replicates, confirming the activity of these compounds in the primary screen. To determine whether hits identified in the first-round screen activated the thermogenic program, *UCP1* transcript levels were used as a readout. This secondary screen incorporated adrenergic agonists, such as Mirabegron, isoproterenol, and phenylephrine, and 17 additional compounds with no reported function in thermogenic regulation (Fig. 6e and Supplementary Fig. 9). In all, 17/24 (71%) of the lipolysis-activating compounds identified in the primary screen also activate the thermogenic program through *UCP1*. Eleven of these compounds have no reported adrenergic agonist activity and could potentially operate through different signaling mechanisms (Fig. 6e and Supplementary Data 1). Of note, two phosphodiesterase III (PDE III) inhibitors were identified as activators in both rounds of screening. Other classes of compounds include receptor/channel antagonists (L-368,899, pilocarpine nitrate, LY310-762, Felodipine) and protein kinase inhibitors (Dinaciclib, AT9283, PF-04691502, PIK-75, SU 5416). These data demonstrate the broad utility of beige adipocytes as a platform for drug discovery and hit/target validation studies.

## Discussion

Current technologies for generating thermogenic adipocytes are limited by their reliance on genetic modifications^18^, low efficiency owing to the use of serum and embryoid bodies^19,20^. Moreover, there is an absence of data regarding the functional validation of these cells in animal models^21^. The use of thermogenic adipocytes derived from induced pluripotent stem cells for autologous transplantations is also not currently feasible owing to low purity and lengthy reprogramming and/or differentiation protocols^16–18^. The technology described in this report solves many of the existing problems associated with the use of human thermogenic adipocytes for therapeutic development. Utilizing a fully defined, serum-free differentiation medium in conjunction with ADSCs allows for highly efficient generation of functional beige adipocytes over a time-frame that is compatible with therapeutic development. Previous studies indicate that diabetic ADSCs lack beige adipogenic potential^21^. This could be circumvented by reprogramming to an iPSC state, followed by differentiation to beige cells^21^. This study indicates that ADSCs derived from diabetic and non-diabetic patients have similar beige differentiation capacity (Supplementary Fig. 8c, Lot F4521). Although there is some variability between ADSCs from different donors, all cell batches tested were capable of generating UCP1^+^ cells at high efficiency. The variations observed can be accounted for by biological variation between donors, as described for other stem/progenitor cells^46^. It was also noted that late passage ADSCs (p10) generated beige cells with higher basal levels of *UCP1* transcript compared with lower passage cells (p5). This could be related to the proliferative potential of ADSCs at different passage numbers or to increased adaptation times in culture.

The ADSC-derived beige adipocytes described in this report exhibit a molecular profile that is consistent with them being authentic beige adipocytes. Moreover, these cells have metabolic characteristics consistent with them having thermogenic activity, including FSK-responsive uncoupled respiration and lipolysis. Using rodent transplantation models, ADSC-derived beige adipocytes increase whole-body energy consumption, resulting in decreased body mass and improved glucose homeostasis. These characteristics establish human ADSC-derived beige adipocytes as having potential utility in the treatment of metabolic disease. Further larger-scale studies are needed to understand how beige adipocyte transplantations can be utilized to promote weight loss. However, as body mass composition analysis can often lead to erroneous analyses in rodent models^47^, the exact nature of the weight loss (reduced fat mass versus reduced bone density) is more difficult to determine. In such cases, larger animal models such as non-human primates like Rhesus macaques may be more suitable.

An additional advantage that ADSC-derived beige cells have over the use of pluripotent cells is that the former can be easily isolated from liposuction aspirates of peripheral white adipose. This can potentially generate a large supply of beige adipocytes for autologous transplantation in a relatively short time-frame, alleviating problems associated with genomic drift during amplification and thereby providing a safe, transplantable cell source. Over 150 clinical trials have described the efficacious and safe use of ADSCs (clinicaltrials.gov), thereby demonstrating their utility in a therapeutic setting. The therapeutic pipeline that we envisage for ADSC-derived beige cells would only require an additional differentiation procedure prior to transplantation. As an alternative approach, an “off the shelf” allogeneic beige adipocyte cell therapy could be developed using immune tolerant scaffolds^48,49^.

Use of beige adipocyte in a transplant setting could potentially follow a similar approach used for β-cell and pancreatic progenitor cell transplants into T1D patients, where encapsulation in a protective device is used^50,51^. The omental pouch^52^ and subcutaneous sites^50^ have been proposed as suitable transplantation sites owing to their potential for neo-vascularization around grafts. High-efficacy has been reported for islet transplants into the omental pouch in diabetic rodent models, canine models, and non-human primate models^53–57^ and subcutaneous transplantation of devices into patients is in clinical trials^50,51^. Both represent potential sites for beige cell transplantations.

Assays of transplanted human beige cells following transplantation showed they maintained high *UCP1* expression, resulting in increased EE and ^18^F-FDG uptake. This can be explained by the maintenance of basal thermogenic activity, as was observed in vitro, or responsiveness to physiological cues such as β-adrenergic signaling through the sympathetic nervous system. The latter scenario would require that transplanted adipocytes be innervated by β-adrenergic neurons.

A wide-range of drugs are currently used for the treatment of T2D but none specifically target thermogenic adipocytes as a means of controlling EE and circulating levels of glucose and triglycerides. A desired effect for T2D drugs is to normalize levels of circulating blood glucose (A1C < 7.0). This can be achieved by targeting different cell types and regulatory mechanisms and where treatment is dependent on drug efficacy and contraindications^1^. Most T2D drugs, however, have restricted target activity and efficacy thus highlighting the need to develop additional therapeutic approaches. Metformin, the first-line orally administered therapeutic for T2D, primarily functions by inhibiting gluconeogenesis in the liver^2^ but does not specifically target thermogenic adipocytes, insulin secretion or carbohydrate absorption. In contrast, sulfonureas, dipeptidyl-peptidase inhibitors and meglitinides modulate glucose levels by promoting insulin secretion. Another approach used clinically is to administer inhibitors of α-glucosidases, which decrease intestinal carbohydrate digestion and absorption rates^2^. Nearly all of these drugs have potential side-effects including weight gain, gastrointestinal effects and hypoglycemia^2^ and importantly, none of them target thermogenic adipocytes. Development of drugs targeting thermogenic adipocytes could have widespread application among T2D patients and for the prevention of disease progression in pre-diabetics (A1C: 5.7–6.4), where there are no FDA-approved medications as a sole-treatment option^1^. The proof-of-concept drug-screening described in this study make drug development in this area feasible.

Identification of multiple β-adrenergic agonists in the HTS validated the cell platform being used for further library screening and therapeutic development. Although β3-adrenergic receptor (β3-AR) agonists can potentially target human thermogenic adipocytes, this idea is primarily based on murine studies^17,58,59^. The role of β-adrenergic signaling is less clear in humans^60^, partly because β3-AR agonists show no significant impact on EE in clinical trials^61–63^. Only one agonist linked to β3-AR signaling (Mirabegron) was shown to have efficacy in the HTS while 35 β1-AR, β2-AR or α-AR agonists were identified. Although β1-AR and β2-AR agonists show efficacy, they are unlikely to have utility for the treatment of T2D because of their known roles in regulation of cardiovascular function^51^. The same may also be true for β3-AR agonists because the β3-AR is expressed in a broader range of tissues than initially thought^64^. These points raise questions about the use of β-adrenergic agonists to target brown and beige adipocytes in a clinical context. The identification of non-β-adrenergic mechanisms that activate thermogenic adipocytes also highlights the need to identify new targets and develop alternative strategies for development of therapeutics that target brown and beige cells^65,66^. HTS using beige cells is likely to be a viable approach to identify drugs that can address some of the therapeutic problems associated with treatment of T2D. In total, 12 compounds were identified that activated the thermogenic program, as determined by *UCP1* induction and lipolytic activity. There is no evidence however, that links these compounds to β-adrenergic signaling so they potentially impact thermogenic adipocytes through non-β-adrenergic mechanisms. Most surprising was the identification of PDE III inhibitors (trequinsin and anagrelide) as having efficacy in beige cell activation. Links between PDE III and the activity of thermogenic adipocytes have not been reported, although inhibition of PDE IV in mouse primary brown adipocytes activates thermogenesis through a PKA-dependent mechanism^67,68^. No links between these compounds and metabolic disease have been established, indicating that use of ADSC-derived beige adipocytes has significant promise as a platform for drug discovery in this area. Further testing is now required to establish the efficacy of compounds identified here in the modulation of metabolic activity in vivo.

## Methods

A step-by-step protocol describing the generation of beige adipocytes from ADSCs can be found at Protocol Exchange (ref. ^69^).

### Cell culture and differentiation of adipose-derived stem/stromal cells

Human adipose-derived stem/stromal cells (ADSCs) (Thermo Fisher, cat no: R7788115, Lot#: 1001001 and Lot #1001002; ATCC, ASC52telo, cat no: ATCC SCRC-4000) were grown in ADSC-growth medium composed of 10% fetal bovine serum (Atlanta Biologicals, S10250) in DMEM (Corning, 10013CV) with 1× Antibiotic-Antimycotic (Corning, 30-004-CI), 1× MEM non-essential amino acids (Corning, 25-025-CI), 1× Glutagro (Corning, 25-015-CI) and 1× BME (Thermo Fisher, 21985023). Except where otherwise indicated, Lot #1001002 was used for the assays. Cells were seeded at a density of 5000 cells per cm^2^ and passaged at 80–90% confluency, at ~5 days post plating. To passage cells from a 100 mm cell culture plate, ADSCs were washed with DPBS (Corning, 21031CV) and then incubated with 5 ml of Accutase (Innovative Cell Technologies, AT104) for 5 minutes at room temperature. Next, 5 ml of DPBS was added and cells were centrifuged at 200 × *g* for 4 minutes in a swinging bucket centrifuge. Cells were resuspended in ADSC-growth medium and counted using a hemocytometer for seeding. Following cell seeding, media was changed every second day. Additional ADSC lines purchased from Lonza, PT-5006 (Lot 0000543947 and 18TL215666); PT-5008 (1F4521 and 1F4619) were grown using the ADSC Bulletkit and ReagentPack (PT-4505 and CC-5034) according to manufacturer’s instructions.

Beige adipocyte differentiation of ADSCs was performed using B-8 medium; a formulation comprising a chemically defined based medium (DM) supplemented with eight growth factors or inhibitors. DM was comprised of DMEM/F-12 w/o glutamine supplemented with 2% Probumin Bovine Serum Albumin (EMD Milipore, 821005), 1× Antibiotic-Antimyotic (Corning, 30-004-CI), 1× MEM non-essential amino acids (Corning, 25-025-CI), 1× Trace Elements A (Corning, 99-182-CI), 1× Trace Elements B (Corning, 99-175-CI), 1× Trace Elements C (Corning, 99-176-CI) 50 μg/mL ascorbic acid (Sigma, A8960), 10 μg/mL Transferrin (Athens Research and Technology, 16-16-A32001-LEL), 0.1 mM 2-mercaptoethanol (Gibco, 21985023) and 1× Glutagro (Corning, 25-015-CI). B-8 medium was generated by adding the following eight supplements to DM, 200 ng/mL LONG R3 human IGF-I (Sigma, 85580 C), 8 ng/mL human basic-FGF (R&D Systems, 4114-TC), 100 ng/mL human-BMP7 (R&D Systems, 354-BP/CF), 10 μM Y27632 (R&D Systems, 1254/50), 2 μM Rosiglitazone (R&D Systems, 5325/50), 1 nM Triiodo-L-thyronine (Sigma, 64245-250MG), 1 μM Dexamethasone (R&D Systems, 1126/100) and 500 μM Isobutylmethylxanthine (Sigma, I5879-5G). To generate beige adipocytes, ADSCs were seeded at a density of 5 × 10^3^ cells per cm^2^ and grown to confluency over ~5 days. B-8 medium was subsequently added and medium was changed every other day for 3 weeks.

To generate WA, ADSCs seeded at a density of 5 × 10^3^ cells per cm^2^ were grown to confluency and differentiated using the StemPro adipogenesis differentiation kit (Thermo Fisher, A10070-01). Media was changed every 2 days for 3 weeks.

### Animals

NOD/SCID mice (NOD/ShiLtSz genetic background, Jackson Laboratory, stock no. 001303) were purchased and used to establish a breeding colony. Mice were housed in Tecniplast GM500 individually ventilated cages in a temperature-controlled room with 12 hour light:12 hour dark cycles. 12-week-old female mice were used in the indirect calorimetry assay. Ages and gender for other animals used is specified in the relevant sections below.

Animal experiments were performed following IACUC guidelines at the University of Georgia accredited through AAALAC international. These experiments are in compliance with Public Health Service policy through NIH Office of Laboratory Animal Welfare and USDA Animal Welfare Act and Regulations.

### Immunostaining and flow cytometry

Before fixation, live cells were treated with MitoTracker Deep Red FM (Thermo Fisher, M22426) for 45 minutes in a cell culture incubator. Next, cells were washed in DPBS three times and fixed in 4% formaldehyde (Thermo Fisher, 28906) in DPBS solution for 15 minutes. Cells were blocked in DPBS-based blocking solution, containing 10% donkey serum (Equitech-Bio, SD30-0500), 0.2% Saponin (EMD Millipore, 558255-100GM) and 0.3 M Glycine (Sigma, G7126-1KG) for 1 hour at room temperature. Primary antibodies UCP1 (Abcam, Ab10983) or Perilipin-1 (Cell Signaling Technology, D1D8) were prepared in DPBS-based primary antibody incubation solution containing 10% Donkey serum and 0.2% Saponin at dilutions of 1:500 and 1:200, respectively, and incubated overnight at 4 °C. After 3 × 5-minute washes in 0.2% Saponin-DPBS solution, cells were incubated with secondary antibodies, Alexa Fluor 555 donkey anti-Rabbit IgG (Thermo Fisher, A31572) in DPBS-based secondary antibody incubation solution containing 2.5% Donkey Serum and 0.2% Saponin at dilutions of 1:250 for 1 hour at room temperature in the dark. Following secondary antibody incubation, cells were washed twice with DPBS for 5 minutes each and then incubated with HCS LipidTOX Green Neutral Lipid Stain (Thermo Fisher, H34475) at 1:200 dilution in DPBS for 30 minutes. Subsequent to neutral lipid staining, cells were treated with DAPI (Sigma, D9542) for 5 minutes. Following an additional three washes in DPBS, cells were treated with ProLong Gold mounting medium (Thermo Fisher, P36934). Samples were cured for 24 hours at room temperature in the dark and later used for imaging on an Olympus FV1200 confocal microscope. Quantitation of beige adipocytes was performed using manual counting with the Cell Counting tool on ImageJ, following immunostaining with UCP1, DAPI, and LipidTox Green of beige adipocytes. Six independent fields of view were assessed in a three-step quantitation process. First, every DAPI signal was counted to determine the total number of cells in each field. Second, the UCP1 and LipidTox Green and DAPI channels were overlaid. Importantly, a conservative approach was applied here as dim staining was considered ‘negative’ and is likely to underestimate the % of UCP1^+^ cells. Third, the total number of UCP1^+^ LipidTox Green^+^ double positive cells (DAPI^+^) were used to calculate the total percentages.

Flow cytometry was performed on ADSCs, by incubating 0.5 × 10^6^ cells for 30 minutes at 4 °C with the following antibodies: CD73-BV421 (BD Biosciences, 562431), CD105-BV421 (BD Biosciences, 563920), IgG1 Isotype-BV421 (BD Biosciences, 562438), CD45-PE (BD Biosciences, 555483), IgG1 Isotype-PE (BD Biosciences, 555749), CD34-PE (R&D Systems, FAB7227P), CD90-APC (eBiosciences, 17-0909-42). Cells were washed in PBS and analyzed on a Beckman Coulter CyAn.

### Immunoblotting

To prepare whole-cell lysates for immunoblotting, ~2 × 10^7^ cells were washed with cold DPBS and harvested by scraping with 300 μl cold radioimmunoprecipitation assay (RIPA) buffer (Sigma, R0278) containing 2% SDS (KD Medical, RGE-3235), 1× Complete, Mini, EDTA-free protease inhibitor cocktail (Roche, 11836170001) and 1× phosphatase inhibitor cocktail set II (EMD Millipore, 524625). Cell lysates were heated for 10 minutes at 95 °C and centrifuged at 13,000 × *g* for 10 minutes at 4 °C. Supernatants were removed carefully to avoid any lipid precipitates and transferred to a new tube. Protein concentration was determined using the RC DC protein assay kit I (Bio-Rad, 5000121). Samples (25 μg total protein) were mixed with 2× Laemmli sample buffer (Bio-Rad, 1610737) and loaded onto a Bolt 4–12% Bis-Tris polyacrylamide gel (Thermo Fisher, NW04122BOX). Samples were electrophoresed for 40 minutes using Bolt MES SDS Running buffer (Thermo Fisher, B0002) and blotted onto a 0.45 μm PVDF membrane (Bio-Rad, 1620260) for 1 hour in Bolt transfer buffer (Thermo Fisher, BT00061). Membranes were blocked in 5% non-fat dry milk (Bio-Rad, 1706404) in 1× Tris-buffered saline (Thermo Fisher, BP2471) with 0.05% Tween-20 (Sigma, P7949) (TBST). Membranes were incubated with primary antibody at 1/1000 dilution, overnight at 4 °C in blocking solution. Primary antibodies were UCP1 (Abcam, Ab10983), p-HSL^S660^ (Cell Signaling, #4126), HSL (Cell Signaling, #4107), p-CREB^S133^ (Cell Signaling, #9191), p-P38MAPK^T180/Y182^ (Cell Signaling, #9216), P38MAPK (Cell Signaling, #9212), and Cofillin (Santa Cruz, sc-376476 HRP). The following day, blots were washed three times in TBST for 5 minutes each, incubated with secondary antibody, anti-rabbit-HRP or anti-mouse HRP (Dako, P0448; Dako, P0260) for 1 hour in blocking solution and subsequently washed for 5 minutes in TBST prior to developing. Amersham ECL western blotting detection reagent (GE Healthcare, RPN2106) was used for detection purposes.

### Quantitative reverse transcriptase PCR and RNA-seq analysis

Cells were washed with DPBS, lysed directly on the plate and used for RNA isolation with the E.Z.N.A Total RNA Kit (Omega, R6834-02) according to manufacturer’s instructions. RNA quantitation was performed with the Biotek Synergy 2 plate reader and cDNA was synthesized using 1 μg of RNA with the iScript cDNA Synthesis Kit (Bio-Rad, 1708841). The quantitative reverse transcription-polymerase chain reaction (qRT-PCR) was performed on a ViiA7 Real-Time PCR System (Thermo Fisher) using a TaqMan Universal PCR Master Mix (Thermo Fisher, 4324020) according to manufacturer instructions with the following TaqMan primer/probe sets (UCP1, Hs01084772_m1), DIO2, Hs00988260_m1, 18 S rRNA, Hs03928985_g1). Each assay was performed in triplicate and normalized to 18 S rRNA and graphed as mean ±standard deviation.

For RNA-Sequence (RNA-Seq), total RNA samples were isolated using E.Z.N.A Total RNA kit and DNase treated to remove potential genomic DNA contamination (Omega, E1091). RNA samples with a RIN > 9 were processed for RNA-Seq analysis. An average of 30 million paired-end reads with a length of 75 base pairs were generated per library on a NextSeq platform by Georgia Genomic Facility. Reads were mapped to the human genome (hg19) by STAR v2.5.3a using default setting and read counts were obtained in STAR quant-mode^70^. Gene expression analysis was preformed using limma, Glimma and EdgeR in R Studio^71^. Log-2 normalized counts per million reads were used for generating heatmap. The ADSC-derived beige cells were compared to downloaded datasets (GEO: GSE78647, GSE78544, GSE78568, GSE78535, GSE78528, GSE78623, GSE78608, GSE78607, GSE57896, GSE59703; and EBI: E-MTAB2602).

Principal component analysis of adipose-derived stem cells used in this study and other relevant cell types was performed in R package, DESeq2 with its plot PCA function^72^. R package ‘ggplot2’ was used to better visualize the result^73^. The ADSCs were compared to downloaded data sets (GEO: GSM909310, GSE78615, GSE90271, GSE78609, GSE78635, GSE80095, PRJNA449980, PRJNA277616, PRJNA277616, GSE59703, GSE113764, GSE101655).

### Electron microscopy

ADSCs were differentiated to beige adipocytes on four-well Nunc Lab-Tek chamber slides (Thermo Fisher, 177437) for 21 days. Samples were fixed in 2.5% glutaraldehyde (v/v) and 5 mM CaCl_2_ in 0.1 cacodylate buffer and washed in DPBS, then post-fixed for 1 hour in 1% OsO_4_ in cacodylate buffer with 5 mM CaCl_2_ and 0.8% potassium ferricyanide. Samples were dehydrated in acetone, Epon-embedded and sectioned. Sections were then stained with uranyl acetate and lead citrate and TEM analysis was performed on a JEOL 1210 electron microscope. For SEM, analysis was performed on a FEI Teneo instrument.

### Seahorse metabolic assays

ADSCs were plated on XFe24 plates (Agilent) at 5 × 10^3^ cells per cm^2^ and then grown to confluency. Cells were differentiated to beige or WA as described above. The XF Cell Mito Stress Test Kit (Seahorse Bioscience, 103015-100) was used to perform the assay by following the manufacturer’s instructions. In brief, oligomycin (2 μM), FCCP (2 μM), and rotenone/antimycin A (5 μM each) were used during the assay at indicated time points, following analysis based on their titration. One day before the assay, cells were stimulated with FSK (20 μM) for 24 hours in freshly prepared XF assay medium (Seahorse Biosciences, 102353-100) containing 25 mM glucose, 1× Glutagro and 1 mM sodium pyruvate. One hour prior to the assay, cells were washed three times in freshly prepared XF assay medium and incubated in a non-CO_2_ incubator at 37 °C. Following the assay, cells were resuspended in RIPA buffer and protein concentration in lysates was determined by Bradford assay. Data were normalized by protein concentration and analyzed in Wave software (Agilent).

### Lipolysis assay

ADSCs were seeded at 5 × 10^3^ cells per cm^2^ in 12-well plates, grown for 5 days and then induced to differentiate into beige adipocytes for 21 days. Prior to the assay, cells were washed in DPBS twice and then in DMEM supplemented with 2% fatty-acid free BSA (Santa Crus, sc-500949) and Triascin C (5 μM). Cells were treated with FSK (20 μM) for the indicated time periods and supernatant samples were collected. Lipolysis assays were performed with free glycerol reagent (Sigma, F6428-40ML), according to the manufacturer’s instructions.

### Transplantations and thermal imaging

ADSCs or ADSC-derived beige adipocytes differentiated for 21 days were transplanted into hindlimb muscles (at 10 locations on both sides) of SCID mice. Approximately 2 × 10^6^ cells in total were transplanted into each recipient. As an alternative approach, ~2 × 10^6^ beige adipocytes were subcutaneously transplanted into the intrascapular region. Thermal imaging was performed using a FLIR A300 infrared camera with ResearchIR software to detect the mean temperature recording.

### Indirect calorimetry

Transplanted mice (12-week, female) were housed individually in metabolic cages for 5 days before the transplantation and 7 days after the transplantation with access to food and water ad libitum at 21 °C and with a 12:12-hr light:dark cycle. Seven control mice and eight experiment mice were used for the analysis. No statistical differences in EE, oxygen consumption, and respiratory exchange ratio were observed prior to transplantations. Metabolic parameters (VO_2_, VCO_2_, locomotor activity) were measured continuously by an open-circuit indirect calorimetry system (OxyMax, Columbus Instruments) equipped with infrared beam counters. EE was calculated based on the equation: EE = (3.815 + 1.232 × RER) × VO_2_. For histograms, VO_2_, VCO_2_, RER, and locomotor activity were calculated by averaging values for dark and light cycles separately. Core body temperature by rectal thermometry was measured daily and body weight was recorded weekly.

### Streptozocin-induced hyperglycemia

Male NOD/SCID 6-week old mice were intraperitoneally injected with 150 mg/kg STZ in an ABSL2 biosafety hood. One week after STZ administration, non-fasting glucose levels in STZ-treated mice were measured by a OneTouch Ultra 2 Glucose meter. Mice that have non-fasting blood glucose levels exceeding 250 mg/dL were included in the study and semi-randomly separated into control and experimental groups. The grouping ensures that the non-fasting blood glucose levels and body weight have no significant difference before the transplantation.

ADSCs or ADSC differentiated to beige adipocytes for 21 days, were transplanted into hindlimb muscles on both sides with eight mice per cell type. Approximately 2 × 10^6^ cells in total were transplanted for each host. Blood glucose levels were measured at indicated time points and mean values were graphed as box plots to indicate total changes.

### Positron emission tomography imaging

In all, 3.7 MBq of ^18^F-Fluorodexoxyglucose was injected via the tail vein followed by an acquisition scan 1-hour post injection using a GE eXplore Vista small animal PET/computed tomography system. Isoflurane anesthesia was used on mice throughout the injection and imaging processes. A region of interest (ROI) were drawn over the injection sites and the concentration of average radioactivity was determined based on the mean pixel values within the ROI volume and converted to counts per ml per minute (counts/ml/min). Assuming a tissue density of 1 g/ml, the counts/ml/min was converted to counts/gram/min and divided by the injected dose (ID) to obtain an imaging ROI-derived %ID/g. Four mice were used for each experimental group.

### High-throughput drug screening

ADSCs were plated in 384-well plates at a density of 5000 cell/cm^2^ and grown to confluency over ~4 days. Cells were then differentiated to beige adipocytes using B-8 medium for 21 days. All cell culture and incubations in the 384-well format were done at 37 °C and 5% CO_2_. Compounds from the library of pharmacologically active compounds (LOPAC, Sigma) and the Emory Enriched Bioactive Library^44^ (3889 in total) were added at a final concentration of 20 μM. The LOPAC library are composed of 1280 biologically active compounds and impacts most signaling pathways and drug classes, including cell signaling, phosphorylation, cell stress, ion channels, G-protein coupled receptors, hormone, and cell cycle regulators. The Emory Enriched Bioactive Library consisted of 2609 compounds including 1018 FDA-approved compounds (SelleckChem) that affect over 20 signaling pathways. The use of FDA-approved drugs may allow for re-purposing for future therapeutic development. Lipolysis assays were performed after 24 hours of drug treatment by measuring free glycerol released in the 384-well format, using the procedure described above. Absorbance for each compound was normalized to untreated control samples for each plate and calculated as a percent of the control. Cut-offs for “activating” and “inhibiting” compounds were set at twofold. Validation assays were performed for select compounds at a range of concentration doses.

### Statistics and reproducibility

All statistical analysis was performed using GraphPad Prism 8.0 software. Differentiation of ADSCs to beige adipocytes and immunostaining (Fig. 1d, e) is representative of independent experiments performed >10 times. The SEM/TEM (Fig. 1b/1c) was independently repeated twice with similar results. All transplant, histological, and immuno-analysis (Fig. 5g–j) were representative of transplants found in several host recipients. All images in supplementary figures (Supplementary Figs. 3B, 4, 5A, 5C, 7A–C, 8A, 8C, 15C) are representative of multiple fields of view and from at least two independent experiments.

### Reporting summary

Further information on research design is available in the Nature Research Reporting Summary linked to this article.

## Supplementary information

Supplementary information_new

Supplementary Data 1_new

Supplementary Data 2_new

Reporting Summary

Description of Additional Supplementary Files

## Data Availability

The authors declare that all data supporting the findings of this study are available within the article and its supplementary information files or from the corresponding author upon reasonable request. The RNA-seq data described in this report have been deposited in the Gene Expression Omnibus database under accession code GSE125331 [https://www.ncbi.nlm.nih.gov/geo/query/acc.cgi?acc=GSE125331]. The unprocessed blots for Fig. 4g are available in Supplementary Fig. 17. Ct values for qRT-PCR are shown in Supplementary Data 2.
