# Supplementary material for: Human beige adipocytes for drug discovery and cell therapy in metabolic diseases
Source: Nat Commun. 2020 Jun 2;11:2758. doi: 10.1038/s41467-020-16340-3 (PMC7265435; doi:10.1038/s41467-020-16340-3)
Supplement: Supplementary file 4 — Reporting Summary [file 41467_2020_16340_MOESM4_ESM.pdf]

natureresearch

Corresponding author(s): Stephen Dalton

Last updated by author(s): Jan 30, 2020

Reporting Summary

Nature Research wishes to improve the reproducibility of the work that we publish. This form provides structure for consistency and transparency in reporting. For further information on Nature Research policies, see Authors & Referees and the Editorial Policy Checklist .

Statistics

For all statistical analyses, confirm that the following items are present in the figure legend, table legend, main text, or Methods section.

n/a	Confirmed	
☐	☒	The exact sample size (*n*) for each experimental group/condition, given as a discrete number and unit of measurement
☐	☒	A statement on whether measurements were taken from distinct samples or whether the same sample was measured repeatedly
☐	☒	The statistical test(s) used AND whether they are one- or two-sided<br>*Only common tests should be described solely by name; describe more complex techniques in the Methods section.*
☒	☐	A description of all covariates tested
☒	☐	A description of any assumptions or corrections, such as tests of normality and adjustment for multiple comparisons
☐	☒	A full description of the statistical parameters including central tendency (e.g. means) or other basic estimates (e.g. regression coefficient) AND variation (e.g. standard deviation) or associated estimates of uncertainty (e.g. confidence intervals)
☐	☒	For null hypothesis testing, the test statistic (e.g. *F*, *t*, *r*) with confidence intervals, effect sizes, degrees of freedom and *P* value noted<br>*Give P values as exact values whenever suitable.*
☒	☐	For Bayesian analysis, information on the choice of priors and Markov chain Monte Carlo settings
☒	☐	For hierarchical and complex designs, identification of the appropriate level for tests and full reporting of outcomes
☒	☐	Estimates of effect sizes (e.g. Cohen's *d*, Pearson's *r*), indicating how they were calculated

Our web collection on statistics for biologists contains articles on many of the points above.

Software and code

Policy information about availability of computer code

Data collection	No software was used.
Data analysis	Reads were mapped to the human genome (hg19) by STAR v2.5.3a using default setting and read counts were obtained in STAR quant-mode. Gene expression analysis was preformed using limma, Glimma and EdgeR in R Studio

For manuscripts utilizing custom algorithms or software that are central to the research but not yet described in published literature, software must be made available to editors/reviewers. We strongly encourage code deposition in a community repository (e.g. GitHub). See the Nature Research guidelines for submitting code & software for further information.

Data

Policy information about availability of data

All manuscripts must include a data availability statement. This statement should provide the following information, where applicable:

- Accession codes, unique identifiers, or web links for publicly available datasets
- A list of figures that have associated raw data
- A description of any restrictions on data availability

The RNA-seq data described in this report has been deposited in the Gene Expression Omnibus under the ID code GSE125331.

Field-specific reporting

Please select the one below that is the best fit for your research. If you are not sure, read the appropriate sections before making your selection.

☒ Life sciences ☐ Behavioural & social sciences ☐ Ecological, evolutionary & environmental sciences

Life sciences study design

All studies must disclose on these points even when the disclosure is negative.

Sample size	A sample size of n=3 (or more) was used with independent replicates for cell culture experiments. This number of independent replicates is sufficient to determine if the P<0.05, to provide sufficient statistical significance. For statistical significance of mice experiments n=4 (or more), per group was chosen.
Data exclusions	No data was excluded from the study.
Replication	All experiments were replicated by 2 individuals, working independently.
Randomization	For cell samples and mice samples, replicates were randomly selected for control or experimental groups.
Blinding	Blinding was not performed during the data collection of the experiments. However, as data analysis and statistical significance was not determined until after the data collection, blinding would would have no impact on the studies performed.

Reporting for specific materials, systems and methods

We require information from authors about some types of materials, experimental systems and methods used in many studies. Here, indicate whether each material, system or method listed is relevant to your study. If you are not sure if a list item applies to your research, read the appropriate section before selecting a response.

Materials & experimental systems

n/a	Involved in the study	
☐	☒	Antibodies
☐	☒	Eukaryotic cell lines
☒	☐	Palaeontology
☐	☒	Animals and other organisms
☒	☐	Human research participants
☒	☐	Clinical data

Methods

n/a	Involved in the study	
☒	☐	ChIP-seq
☐	☒	Flow cytometry
☒	☐	MRI-based neuroimaging

Antibodies

Antibodies used	UCP-1 (Abcam, Ab10983), Perilipin-1 (Cell Signaling Technology, D1D8), CD73-BV421 (BD Biosciences, 562431), CD105-BV421 (BD Biosciences, 563920), IgG1 Isotype-BV421 (BD Biosciences, 562438), CD45-PE (BD Biosciences, 555483), IgG1 Isotype-PE (BD Biosciences, 555749), CD34-PE (R&D Systems, FAB7227P), CD90-APC (eBiosciences, 17-0909-42), UCP1 (Abcam, Ab10983), p-HSLS660 (Cell Signaling, #4126), HSL (Cell Signaling, #4107), p-CREBS133 (Cell Signaling, #9191), p-P38MAPKT180/Y182 (Cell Signaling, #9216), P38MAPK (Cell Signaling, #9212), and Cofillin (Santa Cruz, sc-376476 HRP), anti-rabbit-HRP (Dako, P0448), anti-mouse HRP (Dako, P0260)
Validation	Each antibody was purchased from a commercial vendor, as indicated. Each vendor provided a data-sheet for their antibody, confirming the utility for the antibody in the application used in this study.

Eukaryotic cell lines

Policy information about cell lines

Cell line source(s)	ADSC cell lines were purchased (ThermoFisher, cat no: R7788115, Lot#: 1001001 and Lot #1001002; ATCC, ASC52telo, cat no: ATCC SCRC-4000; Lonza, cat no: 5006, Lots: 0000543947, 18TL215666; cat no: PT-5008, Lots: 1F4521, 1F4619).
Authentication	ADSC lines were authenticated based on presence/absence of cell surface markers CD73, CD105, CD45, CD34 and CD90
Mycoplasma contamination	All cell lines were confirmed to be negative by mycoplasma testing.
Commonly misidentified lines (See ICLAC register)	No commonly misidentified cell lines were used in this study.

Animals and other organisms

Policy information about studies involving animals; ARRIVE guidelines recommended for reporting animal research

Laboratory animals	NOD/SCID mice (NOD/ShiLtSz genetic background, Jackson Laboratory, stock no. 001303) were 12-week old females for indirect calorimetry or 6-8 week old male for blood-glucose studies.
Wild animals	Study did not involve wild animals.
Field-collected samples	Study did not involve samples collected from the field.
Ethics oversight	Animal experiments were performed following IACUC guidelines at the University of Georgia accredited through AAALAC international. This was in compliance with Public Health Service policy through NIH Office of Laboratory Animal Welfare and USDA Animal Welfare Act and Regulations.

Note that full information on the approval of the study protocol must also be provided in the manuscript.

Flow Cytometry

Plots

Confirm that:

- [x] The axis labels state the marker and fluorochrome used (e.g. CD4-FITC).
- [x] The axis scales are clearly visible. Include numbers along axes only for bottom left plot of group (a 'group' is an analysis of identical markers).
- [x] All plots are contour plots with outliers or pseudocolor plots.
- [x] A numerical value for number of cells or percentage (with statistics) is provided.

Methodology

Sample preparation	ADSCs were collected by first washing cells in PBS, and treating cells with Accutase for 5-10 minutes at room temperture to develop a single-cell suspension. Cells were counted and washed, and aliquoted 0.5 million cells/tube/antibody. Antibody was added to the cells, and incubated at 30 min (or as indicated by antibody vendor work-sheet). Cells were pelleted, washed, and used for analysis.
Instrument	Beckman Coulter CyAn
Software	FlowJo7 was used to analyze the flow cytometry data.
Cell population abundance	Cells were not sorted and only used for analysis. Following analysis, cells were discarded.
Gating strategy	Cells were only gated on FSC/SSC and no further gating strategy was used.

- [ ] Tick this box to confirm that a figure exemplifying the gating strategy is provided in the Supplementary Information.
