# Supplementary material for: Human beige adipocytes for drug discovery and cell therapy in metabolic diseases
Source: Nat Commun. 2020 Jun 2;11:2758. doi: 10.1038/s41467-020-16340-3 (PMC7265435; doi:10.1038/s41467-020-16340-3)
Supplement: Supplementary file 5 — Description of Additional Supplementary Files [file 41467_2020_16340_MOESM5_ESM.pdf]

Title: Supplementary Data 1.
Description: Dose-response and validation assays of compounds identified in the primary screen. Beige adipocytes were differentiated from ADSCs for 21 days in 384-well plates and treated with compounds overnight at indicated doses in quadruplicate. Data are expressed as a percentage of control (DMSO).

Title: Supplementary Data 2.
Description: Average CT values for qRT-PCR data.
