# Supplementary material for: Human beige adipocytes for drug discovery and cell therapy in metabolic diseases
Source: Nat Commun. 2020 Jun 2;11:2758. doi: 10.1038/s41467-020-16340-3 (PMC7265435; doi:10.1038/s41467-020-16340-3)
Supplement: Supplementary file 1 — Supplementary information_new [file 41467_2020_16340_MOESM1_ESM.pdf]

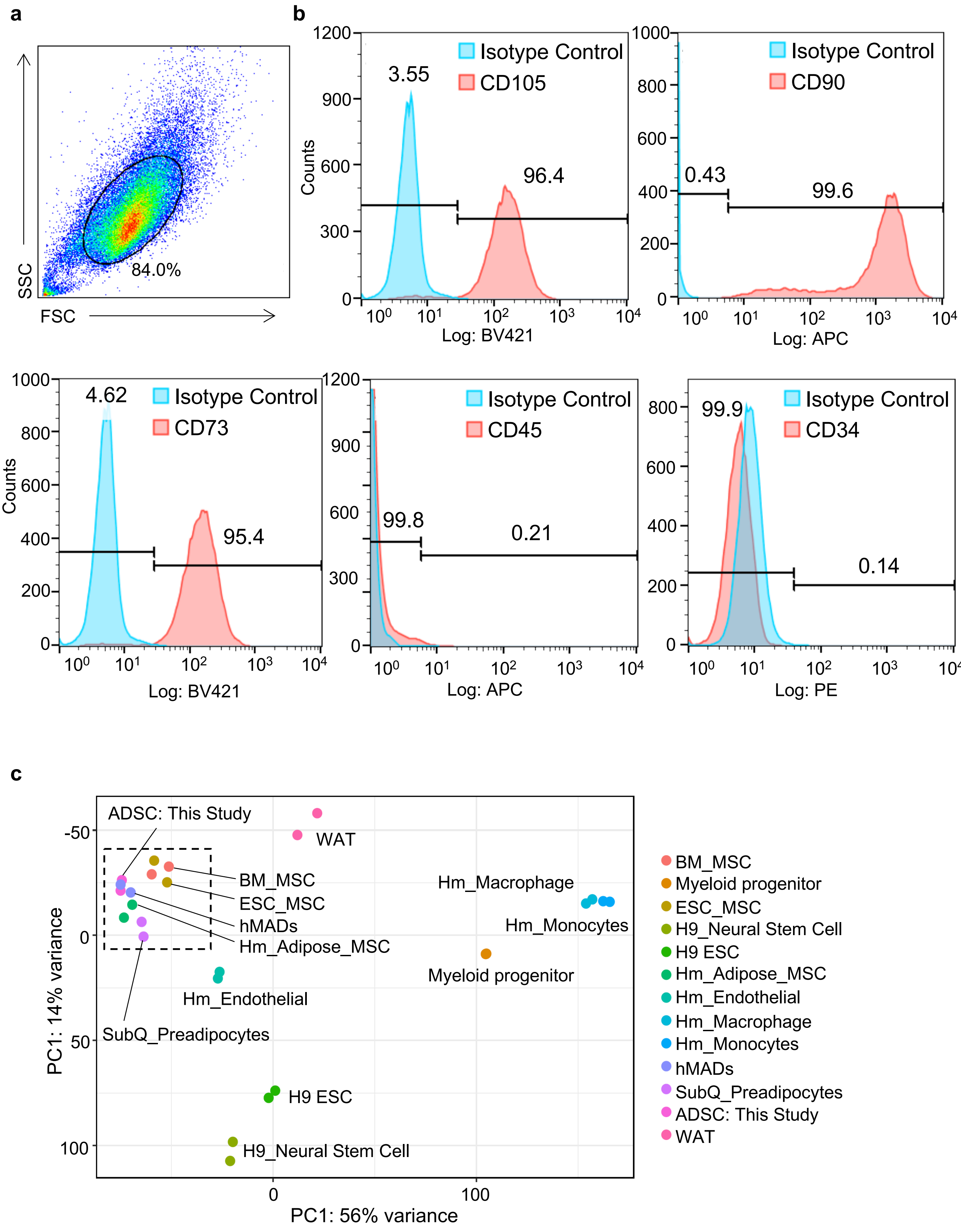
a
SSC
FSC
84.0%
b
Counts
Isotype Control
CD105
3.55
96.4
Log: BV421
Isotype Control
CD90
0.43
99.6
Log: APC
Counts
Isotype Control
CD73
4.62
95.4
Log: BV421
Isotype Control
CD45
99.8
0.21
Log: APC
Isotype Control
CD34
99.9
0.14
Log: PE
c
ADSC: This Study
WAT
BM_MSC
ESC_MSC
hMADs
Hm_Adipose_MSC
Hm_Macrophage
Hm_Monocytes
Myeloid progenitor
Hm_Endothelial
SubQ_Preadipocytes
H9 ESC
H9_Neural Stem Cell
PC1: 14% variance
PC1: 56% variance
BM_MSC
Myeloid progenitor
ESC_MSC
H9_Neural Stem Cell
H9 ESC
Hm_Adipose_MSC
Hm_Endothelial
Hm_Macrophage
Hm_Monocytes
hMADs
SubQ_Preadipocytes
ADSC: This Study
WAT

Supplementary Figure 1. Characterization of ADSCs. (**a**) Gating for flow cytometric analysis of ADSCs was performed on the side-scatter and forward-scatter population. Approximately 0.5 million human adults ADSCs (Thermo Fisher) were immunostained with cell surface markers that characterize mesenchymal cell types for 30 minutes at 4°C and analyzed by flow cytometry. (**b**) Matched isotype control for each antibody was used as a negative control. ADSCs used in this study are characterized as $CD105^{+}$, $CD90^{+}$, $CD73^{+}$, $CD45^{-}$ and $CD34^{-}$. (**c**) PCA plot of ADSCs in this study, compared to mesenchymal stem cells, pre-adipocytes, WAT, macrophage/monocytes, hESCs, endothelial cells and hESC-derived neural stem cells.

a

Factor/small molecule	Enhanced beige differentiation (*UCP1* transcript)	Improved cell viability/survival
IGF1 (200 ng/ml)	+++	+++
FGF2 (8 ng/ml)	+++	+
Y27632 (10 μM)	-	+
BMP7 (100 ng/ml)	++	-
BMP4 (50 ng/ml)	++	-
All-trans retinoic acid (4 μM)	-	-
PD0325901 (1 μM)	-	-
BIO (2 μM)	-	-
CHIR99021 (10 μM)	-	-
LY294002 (50 μM)	-	-
Rapamycin (250 nM)	-	-
SB431542 (20 μM)	-	-

b

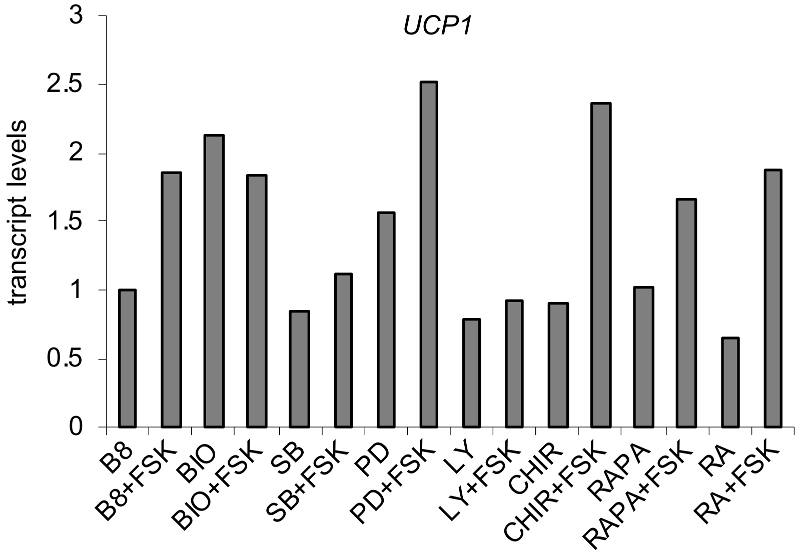

c

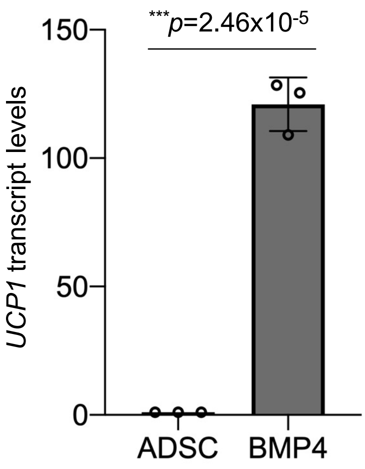

Supplementary Figure 2. Summary of effect of growth factors and small molecules on beige adipocyte differentiation and cell survival. (**a**) Each growth factor or small molecule was added to basal medium (see Supplementary Table 1) with pro-beiging factors (dexamethasone, rosiglitazone, Triiodothyronine and 3-isobutyl-1-methxanthine). IGF1, FGF2 and Y27632 was included with all other factors due to their effect on cell survival. Cells were visually inspected by microscopy and scored, and *UCP1* transcript levels were measured by qRT-PCR. (**b**) Primary qRT-PCR data used for table development. Data generated from technical duplicates. (**c**) BMP4 was substituted for BMP7 in B-8 medium and *UCP1* transcript levels were analyzed by qRT-PCR. Data are presented as mean +/- S.D. and representative of 3 biologically independent replicates. p-values were calculated by unpaired two-tailed Student's t test. n=3.

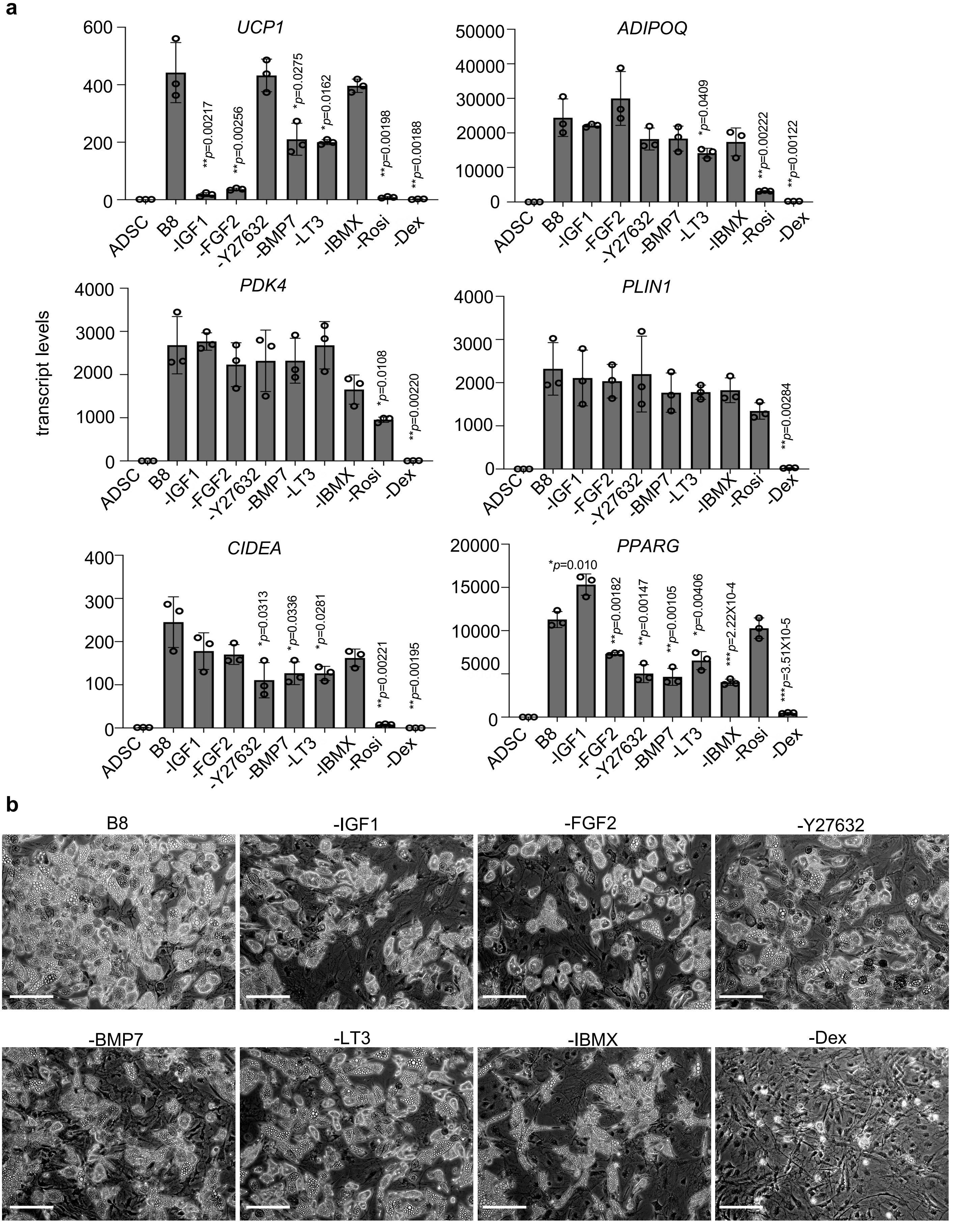

a
UCP1
ADIPOQ
PDK4
PLIN1
CIDEA
PPARG
transcript levels
ADSC
B8
-IGF1
-FGF2
-Y27632
-BMP7
-LT3
-IBMX
-Rosi
-Dex
**p=0.00217
**p=0.00256
*p=0.0275
*p=0.0162
**p=0.00198
**p=0.00188
*p=0.0409
**p=0.00222
**p=0.00122
*p=0.0108
**p=0.00220
**p=0.00284
*p=0.0313
*p=0.0336
*p=0.0281
**p=0.00221
**p=0.00195
*p=0.010
**p=0.00182
**p=0.00147
**p=0.00105
*p=0.00406
***p=2.22X10-4
***p=3.51X10-5
b
B8
-IGF1
-FGF2
-Y27632
-BMP7
-LT3
-IBMX
-Dex

Supplementary Figure 3. Requirement for B-8 factors during beige adipogenesis. (**a**) Transcript analysis of adipocyte markers by qRT-PCR following the removal of each factor during the 21 day differentiation to beige adipocytes. Data are presented as mean +/- S.D. and representative of 3 biologically independent replicates. p-values were calculated by unpaired two-tailed Student's t-test, comparing the indicated "withdrawn-factor" sample to the "B-8" sample. (**b**) Imaging of cells following 21 days of factor withdrawal by phase contrast microscopy. Bar, 50 μm.

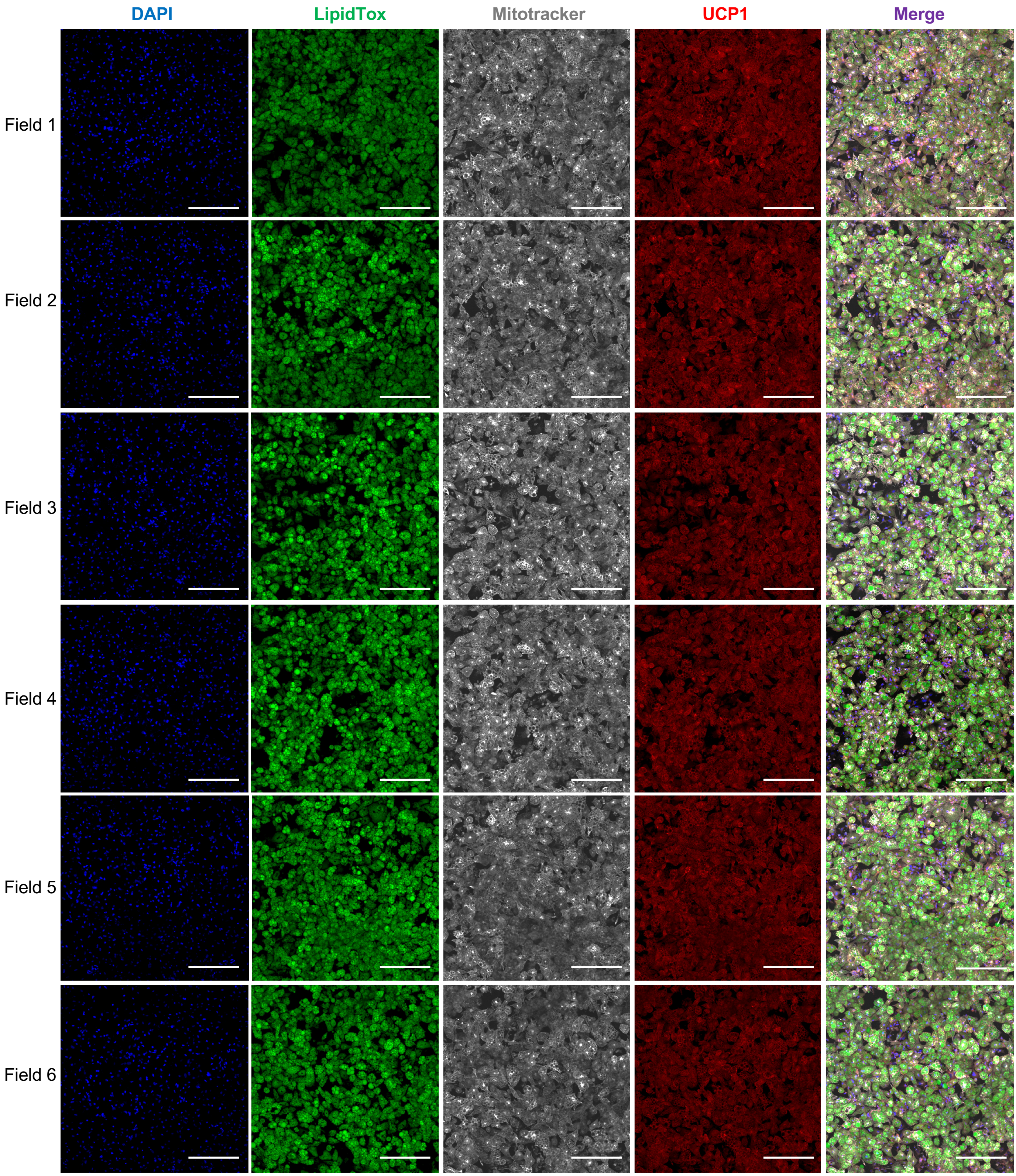

Count Details

Field 1 Total:805 Non-Positive:53
Positive:752 Positive-Percentage:93.4%

Field 2 Total:813 Non-Positive:69
Positive:744 Positive-Percentage:91.5%

Field 3 Total:803 Non-Positive:58
Positive:745 Positive-Percentage:92.8%

Field 4 Total:791 Non-Positive:65
Positive:726 Positive-Percentage:91.7%

Field 5 Total:795 Non-Positive:62
Positive:733 Positive-Percentage:92.2%

Field 6 Total:782 Non-Positive:59
Positive:723 Positive-Percentage:92.4%

Supplementary Figure 4. Quantitation of beige adipocytes in 6 independent fields of view at 10X magnification. The total number of cells was determined by counting DAPI after probing with an anti-UCP1 antibody. Cells that were double-positive for UCP1 and DAPI were scored as beige adipocytes. See methods for a complete description. The percentage of UCP1^{+} cells and the number of cells in each field of view is indicated. UCP1, red; DAPI, blue; Lipid, green. Bar, 200 μm.

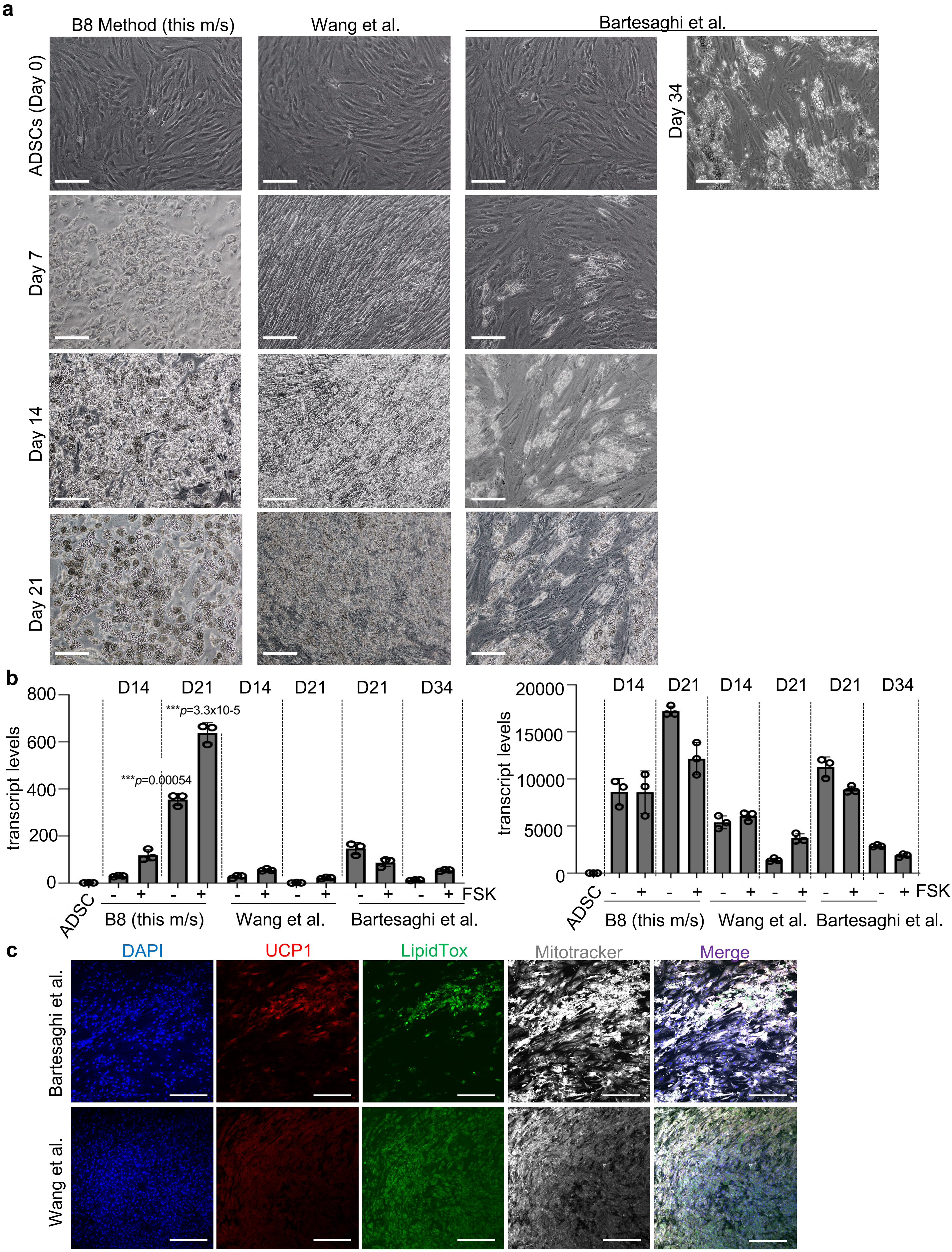
a
B8 Method (this m/s)
Wang et al.
Bartesaghi et al.
ADSCs (Day 0)
Day 34
Day 7
Day 14
Day 21
b
transcript levels
D14
D21
D14
D21
D21
D34
***p=3.3x10-5
***p=0.00054
800
600
400
200
0
ADSC
- + - + - + - + - + - + FSK
B8 (this m/s)
Wang et al.
Bartesaghi et al.
20000
15000
10000
5000
0
c
DAPI
UCP1
LipidTox
Mitotracker
Merge
Bartesaghi et al.
Wang et al.

Supplementary Figure 5. Comparison of B8 method to methods described by Bartesaghi et al[1] and Wang et al[2], respectively. Differentiations were performed exactly as described according to previous publications and imaged; bar: 50 μm. (**a**), and samples were collected for qRT-PCR (**b**) at recommended days. Data are presented as mean +/- S.D. and representative of 3 biologically independent replicates. p-values were calculated by unpaired two-tailed Student's t-test. Statistical significance is shown if B8 method is enhanced over both Bartesaghi et al[1] and Wang et al[2]., (c) Immunostaining of beige cells derived from methods as described by Bartesaghi et al[1] and Wang et al[2] at day 21, bar 200 μm.

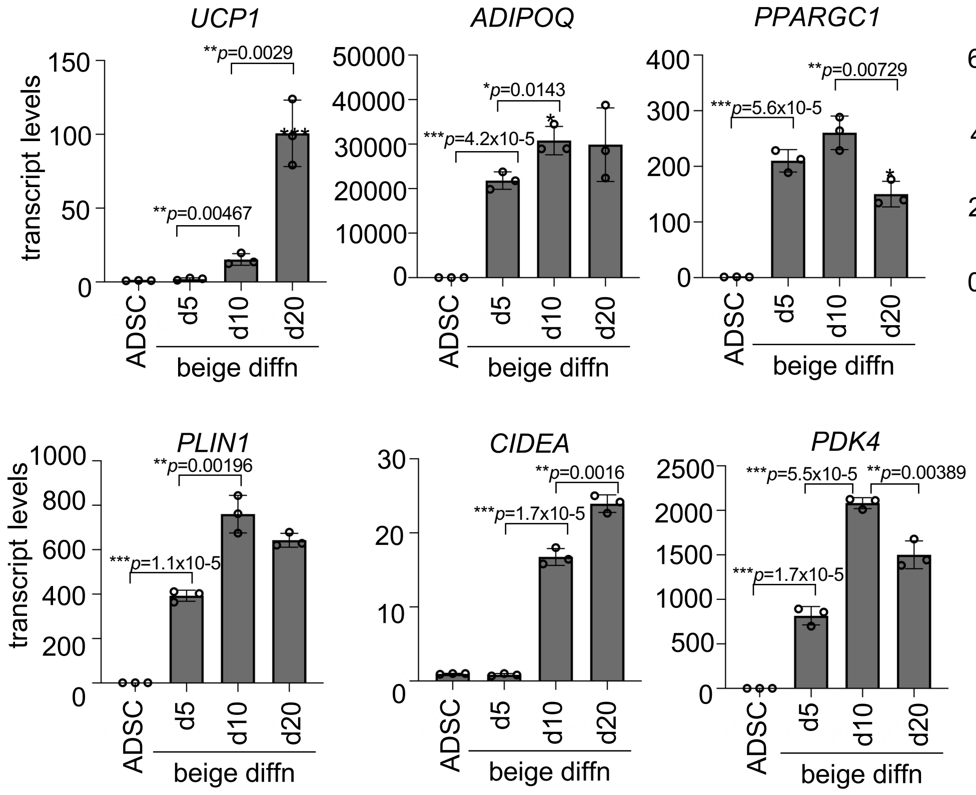

Supplementary Figure 6. Transcriptional analysis of adipocyte markers during beige adipocyte differentiation in B8 medium. Cell lysates were collected at indicated days and RNA was isolated and analyzed by qRT-PCR for marker expression. Each statistical measurement was compared to the preceding time-point (i.e. d5 was compared to ADSC, d10 was compared to d5, d20 was compared to d10). Data are presented as mean +/- S.D. and representative of 3 biologically independent replicates. p-values were calculated by unpaired two-tailed Student's t-test.

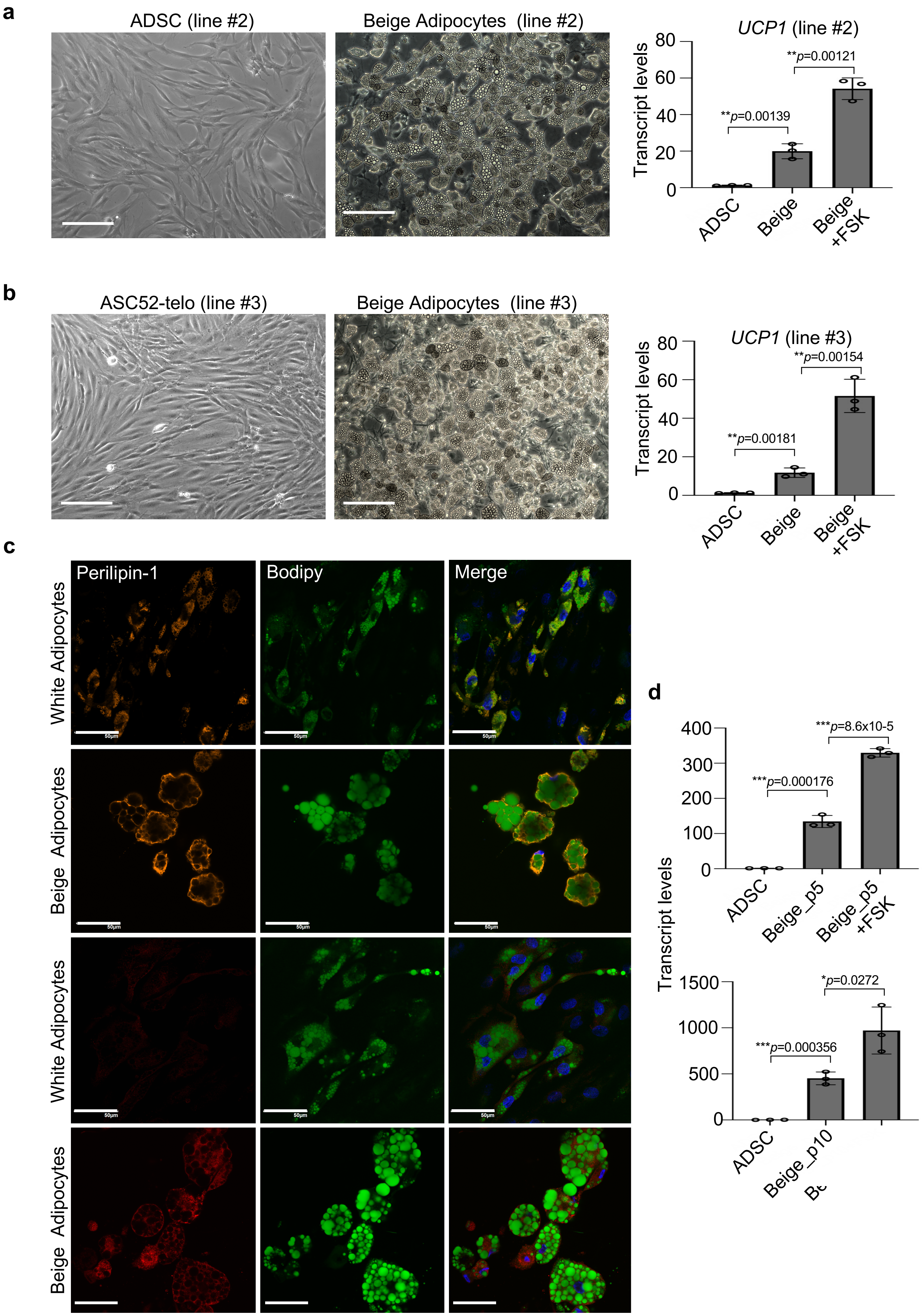
a
ADSC (line #2)
Beige Adipocytes (line #2)
UCP1 (line #2)
Transcript levels
**p=0.00139
**p=0.00121
ADSC
Beige
Beige +FSK
b
ASC52-telo (line #3)
Beige Adipocytes (line #3)
UCP1 (line #3)
Transcript levels
**p=0.00181
**p=0.00154
ADSC
Beige
Beige +FSK
c
Perilipin-1
Bodipy
Merge
White Adipocytes
Beige Adipocytes
White Adipocytes
Beige Adipocytes
d
Transcript levels
***p=0.000176
***p=8.6x10-5
ADSC
Beige_p5
Beige_p5 +FSK
***p=0.000356
*p=0.0272
ADSC
Beige_p10

Supplementary Figure 7. Multiple sources of human ADSCs efficiently generate beige adipocytes. (**a**) A second ADSC isolate also shows high-efficiency differentiation to adipocytes containing lipid droplets and robust induction of *UCP1* transcript levels following forskolin (FSK; 20 μM) treatment. Bar 100 μm. Transcript levels were determined by qRT-PCR. (**b**) An immortalized ADSC line (ASC52-telo) results in high-efficiency differentiation to adipocytes containing multi-locular lipid droplets and robust induction of *UCP1* transcript levels following forskolin (FSK; 20 μM) treatment. Bar, 100 μm. Transcript levels were determined by qRT-PCR in technical triplicate. (**c**) Immunostaining for UCP1 in ASC52-telo cells differentiated to beige adipocytes or ADSC-derived white adipocytes for 21 days, bar 50 μm. (**d**) Transcript levels of *UCP1* of beige adipocytes differentiated from ADSCs (line 1, lot: 1001002) after five passages and ten passages. All qRT-PCR data are presented as mean +/- S.D. and representative of 3 biologically independent replicates. p-values were calculated by unpaired two-tailed Student's t-test.

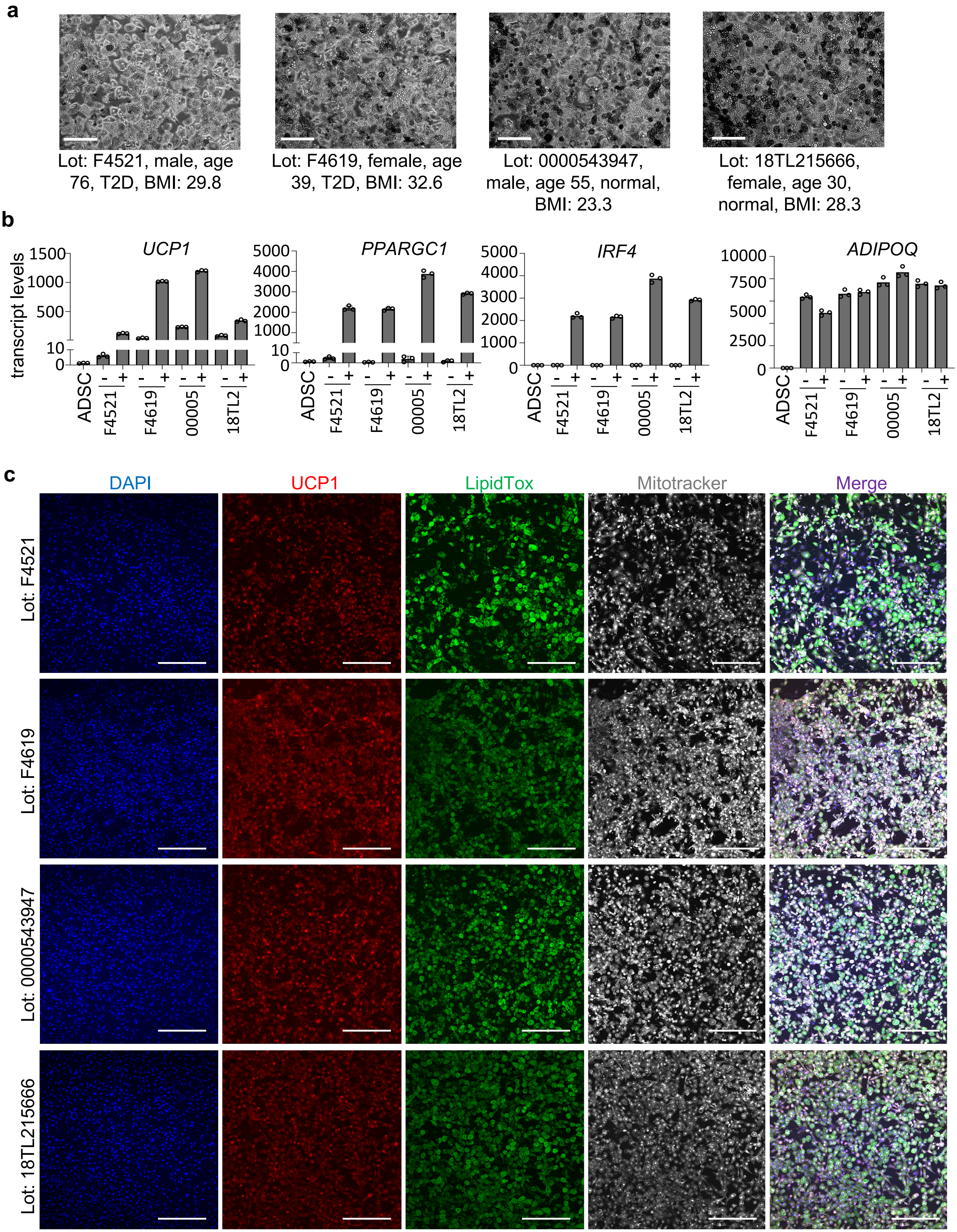
a
Lot: F4521, male, age 76, T2D, BMI: 29.8
Lot: F4619, female, age 39, T2D, BMI: 32.6
Lot: 0000543947, male, age 55, normal, BMI: 23.3
Lot: 18TL215666, female, age 30, normal, BMI: 28.3
b
transcript levels
UCP1
PPARGC1
IRF4
ADIPOQ
0
10
500
1000
1500
1000
2000
3000
4000
5000
2500
5000
7500
10000
ADSC
F4521
F4619
00005
18TL2
-
+
c
DAPI
UCP1
LipidTox
Mitotracker
Merge
Lot: F4521
Lot: F4619
Lot: 0000543947
Lot: 18TL215666

Supplementary Figure 8. Analysis of additional normal and type 2 diabetic (T2D) ADSC lines. Four additional lines (two male and two female) were purchased from Lonza of varying age, BMI and T2D status. Each line was differentiated for 21 days to beige adipocytes using B-8 medium. (a) Phase-contrast images (bar 100 μm), (b) transcript levels determined by Taqman qRT-PCR for indicated genes, +/- FSK for 6 hours (20 μM). Data are presented as mean +/- S.D. and representative of 3 biologically independent replicates. p-values were calculated by unpaired two-tailed Student's t-test. (c) immunostaining using anti-UCP1 antibody following the treatment of FSK for 24 hours. Bar, 200 μm.

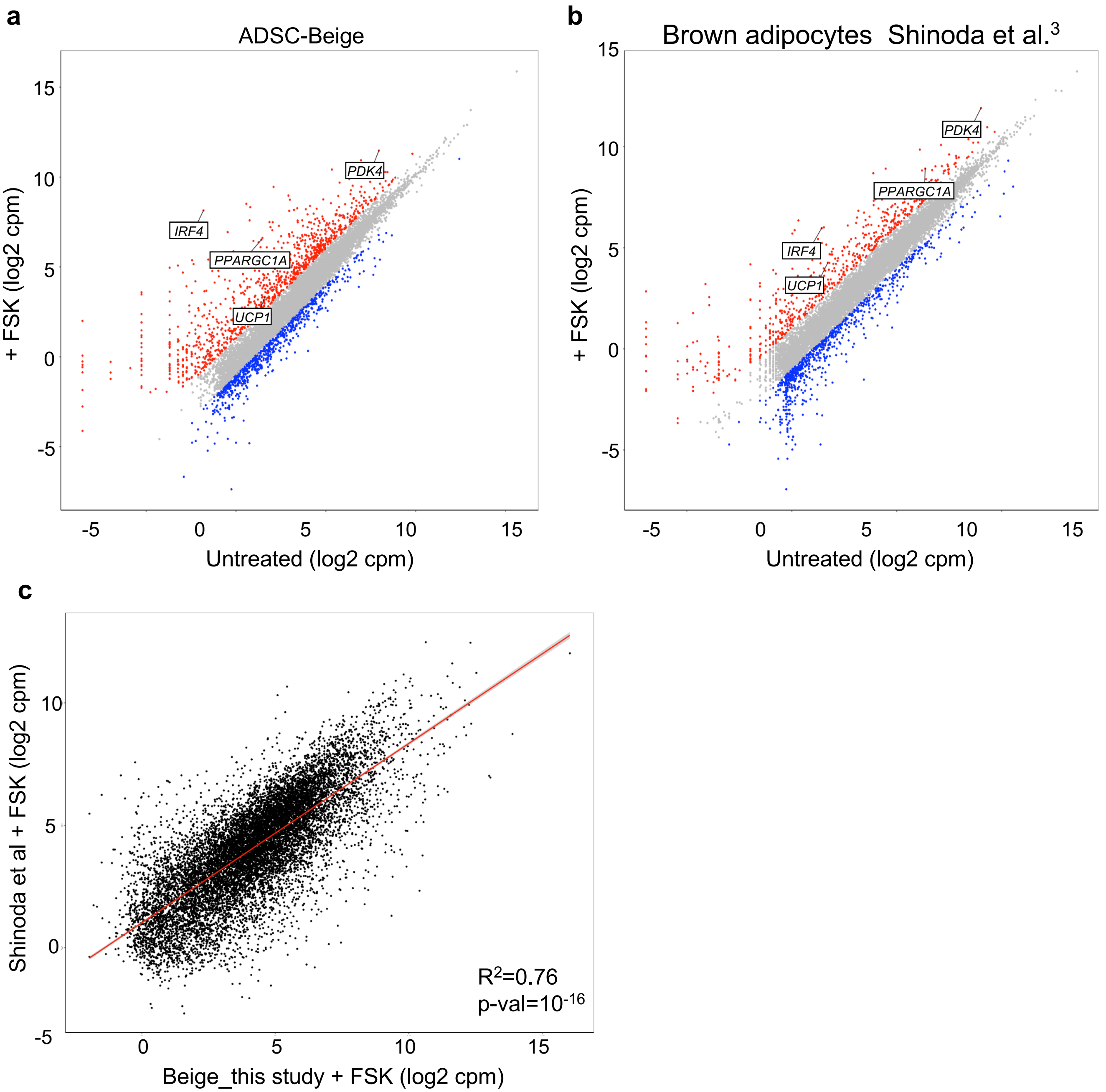

Supplementary Figure 9. Forskolin (FSK) has similar effects on global transcription in beige and brown adipocytes. Scatter plots comparing transcript levels by RNA-seq before and after FSK treatment. (**a**) ADSC-derived beige adipocytes and (**b**) brown adipocytes[3]. Transcripts for thermogenic genes such as *UCP1*, *IRF4*, *PPARGC1A* and *PDK4* are shown to be elevated following FSK (20 μM, 6 hours) treatment. **(c)** Linear regression analysis comparing data from this study with beige adipocytes (FSK-treated and Shinoda et al.[3] FSK-treated, indicating a similar stimulation response.

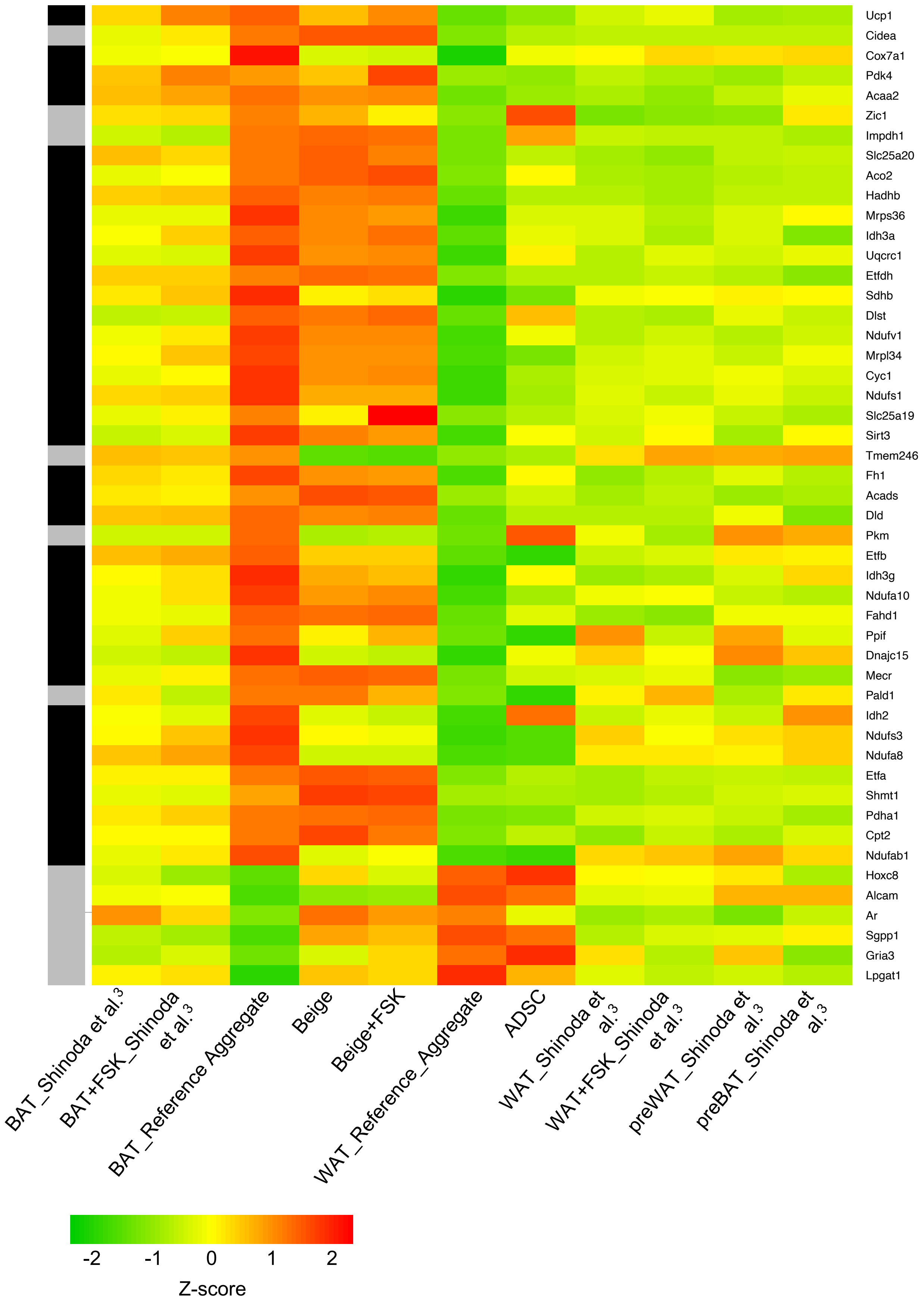
Ucp1
Cidea
Cox7a1
Pdk4
Acaa2
Zic1
Impdh1
Slc25a20
Aco2
Hadhb
Mrps36
Idh3a
Uqcrc1
Etfdh
Sdhb
Dlst
Ndufv1
Mrpl34
Cyc1
Ndufs1
Slc25a19
Sirt3
Tmem246
Fh1
Acads
Dld
Pkm
Etfb
Idh3g
Ndufa10
Fahd1
Ppif
Dnajc15
Mecr
Pald1
Idh2
Ndufs3
Ndufa8
Etfa
Shmt1
Pdha1
Cpt2
Ndufab1
Hoxc8
Alcam
Ar
Sgpp1
Gria3
Lpgat1
BAT_Shinoda et al.3
BAT+FSK_Shinoda et al.3
BAT_Reference Aggregate
Beige
Beige+FSK
WAT_Reference_Aggregate
ADSC
WAT_Shinoda et al.3
WAT+FSK_Shinoda et al.3
preWAT_Shinoda et al.3
preBAT_Shinoda et al.3
-2
-1
0
1
2
Z-score

Supplementary Figure 10. Heatmap from ProFAT[4] analysis comparing indicated samples by adipose transcripts. From a total of 97 human adipocyte RNA-seq and microarray datasets, transcriptional profiles were aggregated together and following the removal of batch effects using a computational algorithm, normalized gene expression values were calculated and used for BAT or WAT references. These BAT and WAT reference aggregates were compared to BA[3] or ADSC-derived beige adipocytes (this study). A total of 49 genes, indicated in the heatmap, were used to interrogate RNA-seq datasets so that a browning probability score was generated (Fig. 2d). Relative gene expression changes are indicated by the Z-score. Black and gray boxes represent predicted mitochondrial or non-mitochondrial genes, respectively.

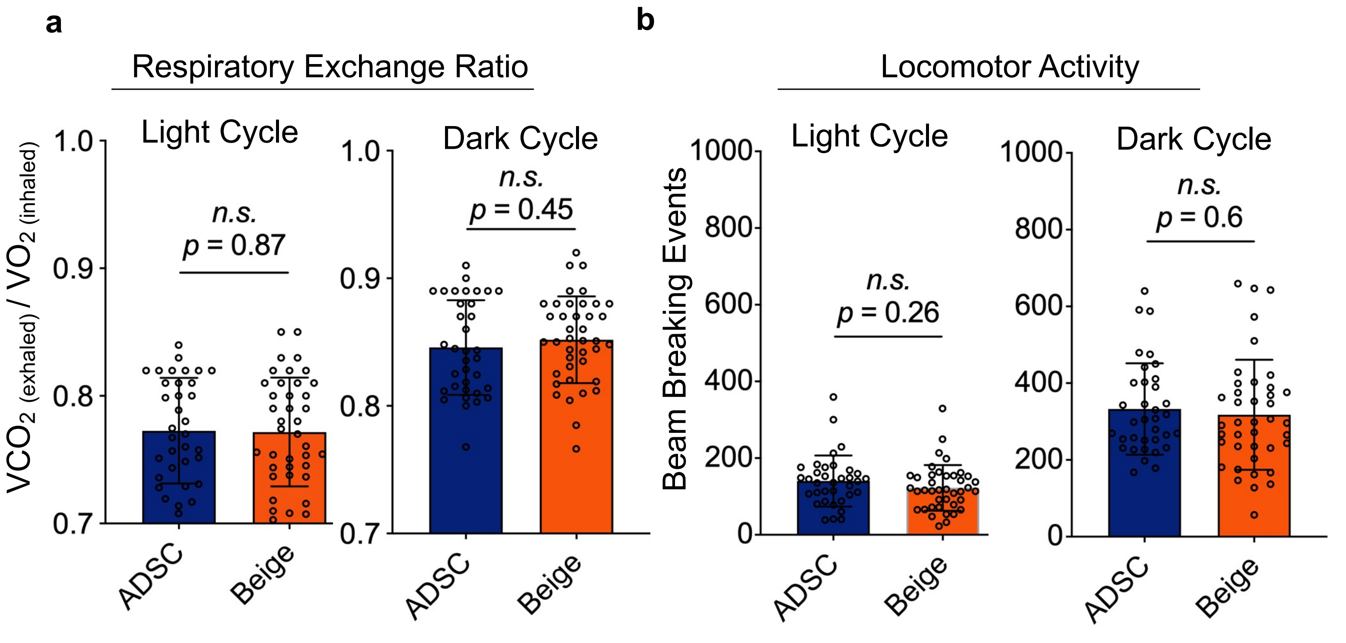

Supplementary Figure 11. Indirect calorimetry assay comparing respiratory exchange ratio and locomotor activity (**a**) Respiratory exchange ratio from indirect calorimetry assay indicate no change between ADSC and beige transplants, suggesting no hyperventilation from the mice. (**b**) Activity levels of mice indicate no change, indicating that increased activity is not the cause for the increased energy expenditure or oxygen consumption; n=7 control, n=8 beige cells.

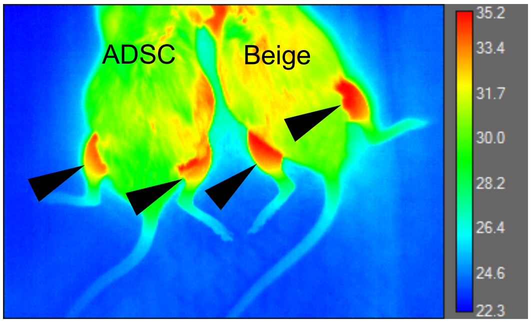

Supplementary Figure 12. Thermal imaging and histology of leg muscles in beige transplanted mice. Representative image of mice transplanted with ADSCs or ADSC-derived beige adipocytes. One week following transplantation, hindlimbs where shaved around the transplantation site and imaged using a FLIR infrared camera. Surface body temperature (°C) was determined as indicated, confirming data in Fig. 4c. Arrowheads depict transplantation sites.

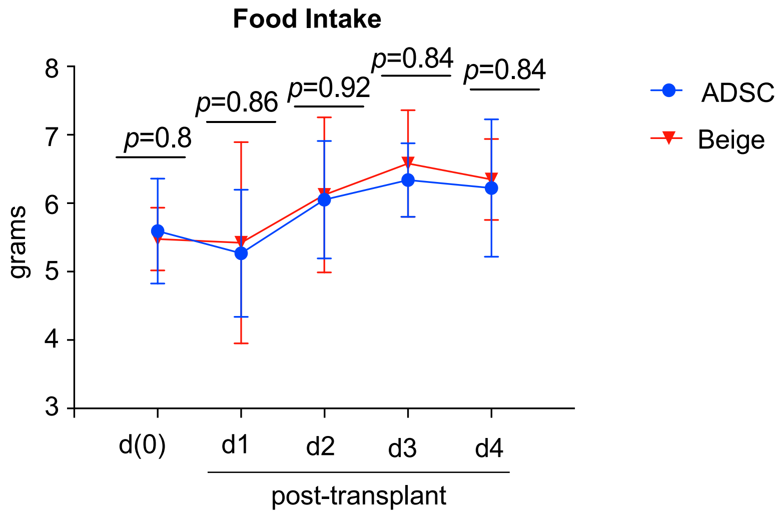

Supplementary Figure 13. Analysis of food intake following beige adipocyte transplantation. Mice (n=5 per group) were transplanted with ADSCs or beige adipocytes into the leg muscle and back and food intake was monitored daily by measuring weight. Data are representative of mean +/- S.D. p-values were calculated by unpaired two-tailed Student's t-test.

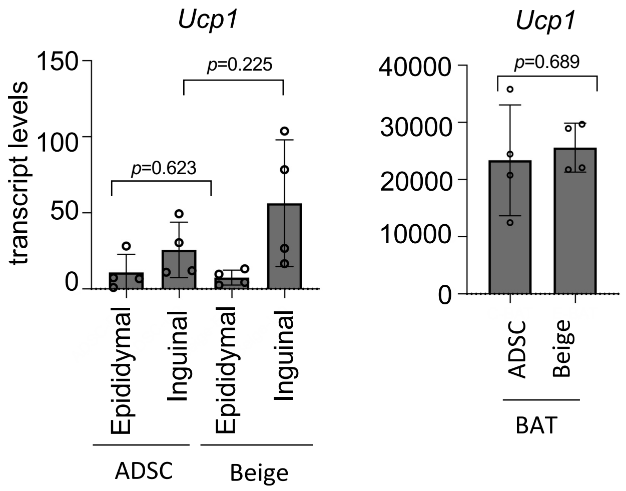

Supplementary Figure 14. Analysis of mouse adipose tissue depots following human beige tissue transplantation. Transcript levels for *Ucp1*, normalized to *Gapdh* were analyzed following transplantation of human beige tissue into the leg and back of NOD/SCID mice by Taqman qRT-PCR per group (ADSC or Beige transplants). Data are presented as mean +/- S.D. and representative of 4 biologically independent replicates. p-values were calculated by unpaired two-tailed Student's t-test.

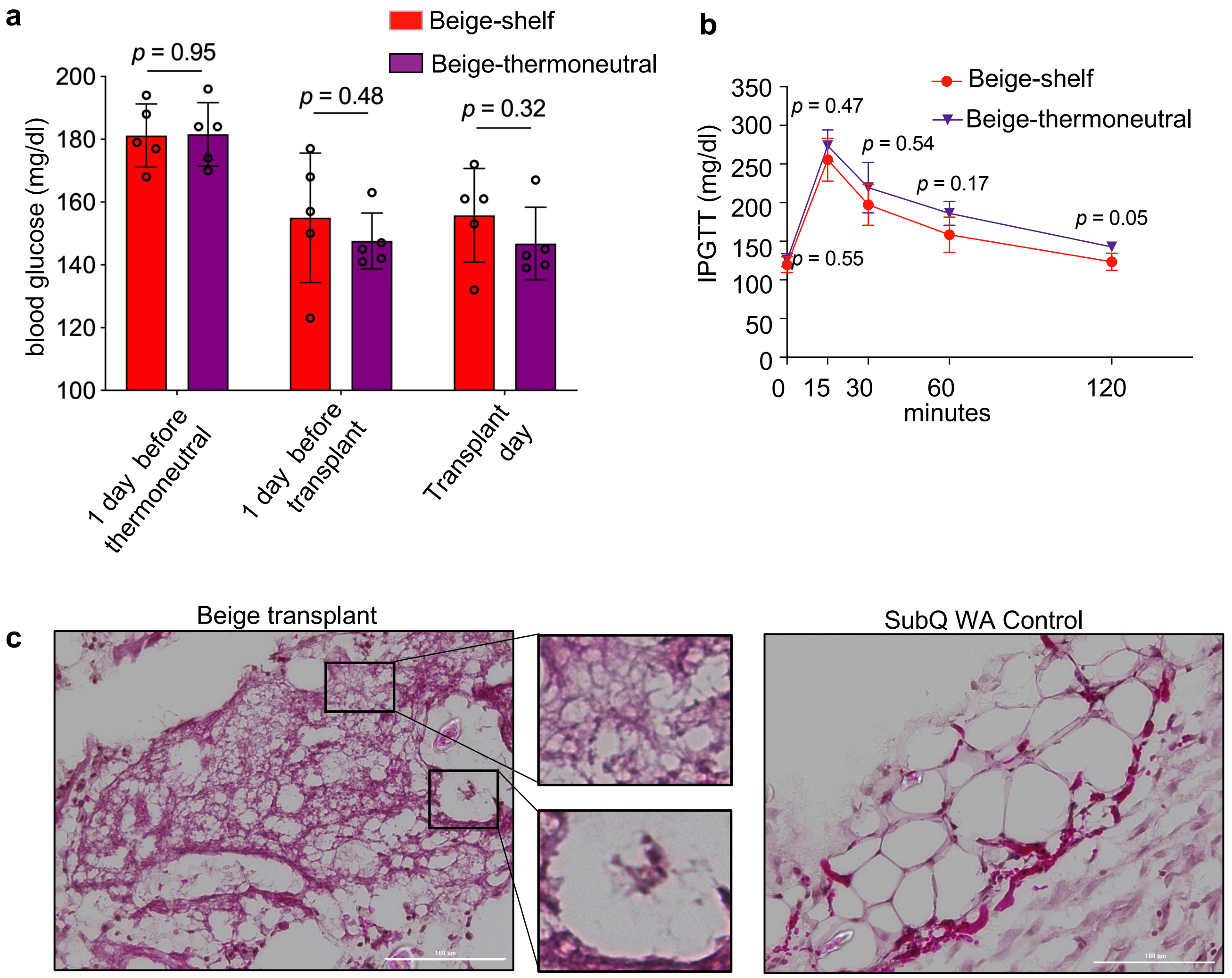

Supplementary Figure 15. Analysis of beige adipocyte transplantation under thermoneutral conditions. Beige adipocytes were transplanted into male 12-week old NOD-SCID mice that were previously housed under standard shelf (21°C) or thermoneutral (30°C) conditions for two weeks, n=5 . Mice were monitored prior to transplant for non-fasting blood glucose levels (**a**), and after 5 days, glucose measurements were taken by IPGTT (**b**). (**c**) Histology analysis of transplants under thermoneutral conditions at day 5 had numerous regions consistent with a "whitening" morphology (compare top and lower panels of magnified regions), bar 100 μm.

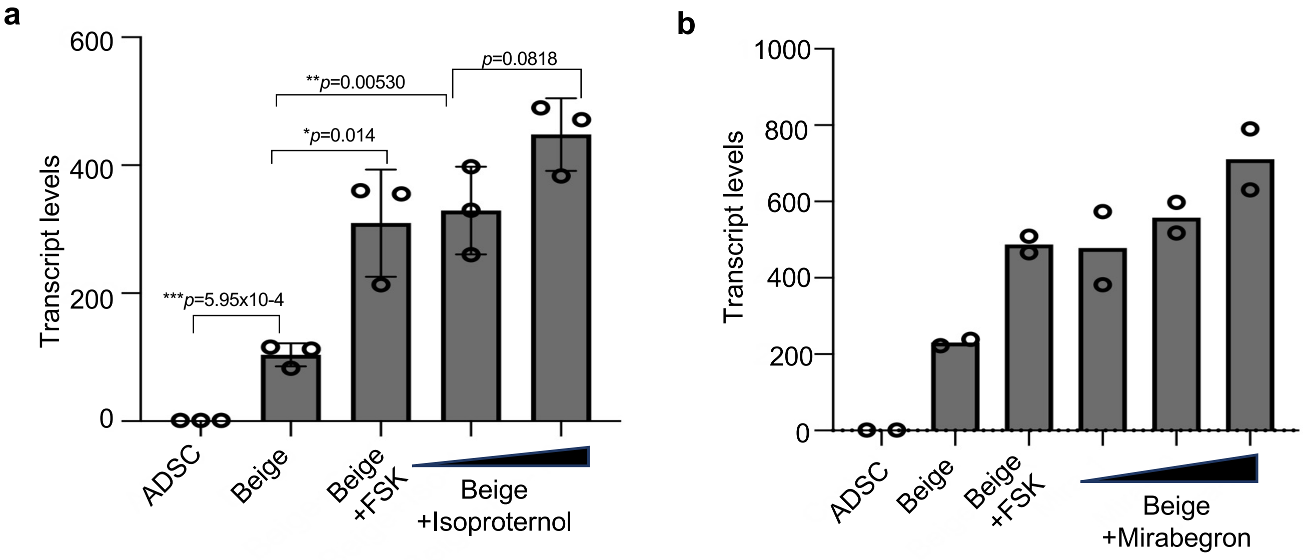

Supplementary Figure 16. Secondary screening of known thermogenic activators. (**a**) Transcript levels of *UCP1* were assayed in unstimulated ADSCs and ADSC-derived beige adipocytes and following treatment with forskolin (FSK, 20 μM) or isoproterenol (1, 10 μM). Transcript levels were determined by qRT-PCR. Data are presented as mean +/- S.D. and representative of 3 biologically independent replicates. p-values were calculated by unpaired two-tailed Student's t-test. (**b**) Transcript levels of *UCP1* were assayed in unstimulated ADSCs and ADSC-derived beige adipocytes and following treatment with forskolin (FSK, 20 μM) or Mirabegron (1, 10 and 25 μM). The assay was performed in biological duplicate.

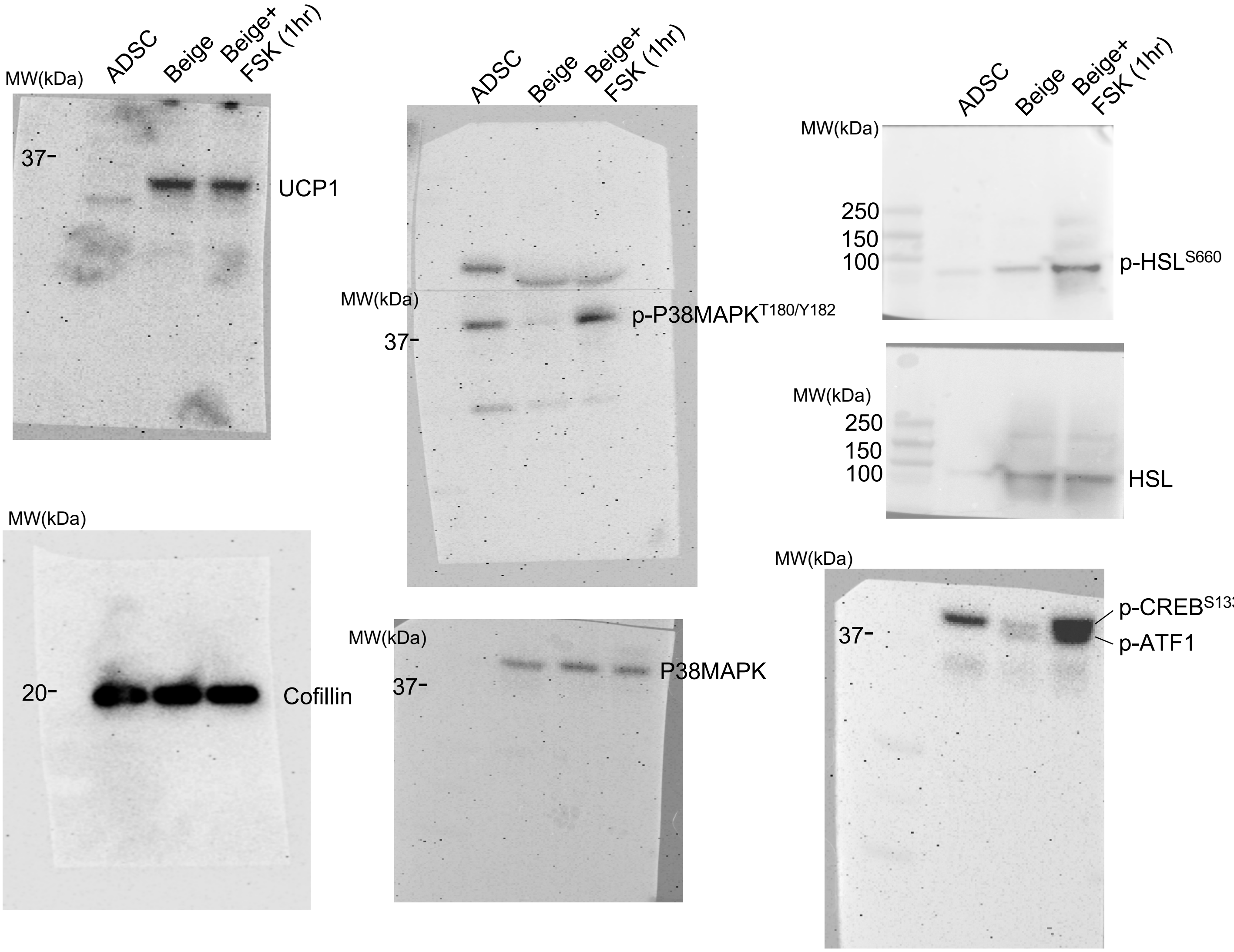

Supplementary Figure 17. Uncropped western blots from Figure 4g. Blots were cut at the 50 kDa marker band. Upper and lower halves of the blot were then probed with different antibodies as indicated.

Supplementary Table 1. Media compositions from previous protocols used to generate ADSC-derived beige adipocytes.

This Study (B-8)	Bartesaghi et al. 2015 (Ref: 1)	Wang et al. 2015 (Ref: 2)
DMEM/F-12	DMEM-F12	α-MEM
Probumin-albumin (2%)	3% fetal calf serum	10% fetal bovine serum
Nonessential amino acids (1X)	Dexamethasone (100 mM)	Dexametasone (100 nM)
GlutaGro (1X)	3-isobutyl-1-methyxanthine (500 μM)	Ascorbate-2-phosphate (50 μg/ml)
Trace Elements A (1X)	insulin (0.85 μM)	insulin (10 μg/ml)
Trace Elements B (1X)	Triiodothyronine (5 nM)	Triiodothyronine (1 nM)
Trace Elements C (1X)	Rosiglitazone (100 nM)	indomethacin (50 μg/ml)
L-ascorbic acid (50 μg/ml)		forskolin (10 μM)
Transferrin (10 μg/ml)		
**IGF-1 (200 ng/ml)**		
**FGF2 (8 ng/ml)**		
**Rosiglitazone (2 μM)**		
**Dexamethasone (1 μM)**		
**Triiodothyronine (1 nM)**		
**BMP7 (100 ng/ml)**		
**3-isobutyl-1-methyxanthine (500 μM)**		
**Y-27632 (10 μM)**		
**Differentiation Efficiency**	**Differentiation Efficiency**	**Differentiation Efficiency**
>90%	Not determined/described	Not determined/described
**NOTE**: Defined base media components are italicized and B-8 factors/inhibitors are bolded		**NOTE**: 3-isobutyl-1-methyxanthine (0.45 mM) was substituted for forskolin in some experiments

Supplementary References

1. Bartesaghi, S. et al. Thermogenic activity of UCP1 in human white fat-derived beige adipocytes. *Mol Endocrinol* **29**, 130-139 (2015).
2. Wang, Y.L., Lin, S.P., Hsieh, P.C. & Hung, S.C. Concomitant beige adipocyte differentiation upon induction of mesenchymal stem cells into brown adipocytes. *Biochem Biophys Res Commun* **478**, 689-695 (2016).
3. Shinoda, K. et al. Genetic and functional characterization of clonally derived adult human brown adipocytes. *Nat Med* **21**, 389-394 (2015).
4. Cheng, Y. et al. Prediction of Adipose Browning Capacity by Systematic Integration of Transcriptional Profiles. *Cell Rep* **23**, 3112-3125 (2018).
